# Modulating Acceptor Phase Leads to 19.59% Efficiency Organic Solar Cells

**DOI:** 10.1002/advs.202413051

**Published:** 2024-12-24

**Authors:** Liang Bai, Sein Chung, Zhenmin Zhao, Jingjing Zhao, Yuqing Sun, Yuan Liu, Lixing Tan, Jiancheng Zhong, Sooji Lyu, Hojun Ji, Kilwon Cho, Zhipeng Kan

**Affiliations:** ^1^ Center on Nanoenergy Research Carbon Peak and Neutrality Science and Technology Development Institute School of Physical Science & Technology Guangxi University Nanning 530004 China; ^2^ Department of Chemical Engineering Pohang University of Science and Technology Pohang 37673 South Korea; ^3^ State Key Laboratory of Featured Metal Materials and Life‐cycle Safety for Composite Structures Nanning 530004 China

**Keywords:** bulk heterojunction solar cells, dual additives, fibrillar morphology, molecular aggregation, NFAs acceptors

## Abstract

Nonfullerene acceptors are critical in advancing the performance of organic solar cells. However, unfavorable morphology and low photon‐to‐electron conversion in the acceptor range continue to limit the photocurrent generation and overall device performance. Herein, benzoic anhydride, a low‐cost polar molecule with excellent synergistic properties, is introduced in combination with the traditional additive 1‐chloronaphthalene to optimize the aggregation of nonfullerene acceptors. This dual additive approach precisely modulates the morphology of various acceptors, significantly enhancing device performance. Notably, the method induces the formation of fine fibers with dense polymorph structures in BTP‐base derivatives, achieving an optimal balance between exciton dissociation and charge collection in the active layers. As a result, the external quantum efficiency of the optimal devices is markedly improved in the wavelength range of 700–850 nm. Ultimately, power conversion efficiencies of 18.27% and 19.59% are achieved for devices comprising PM6:Y6 and PM6:L8‐BO, respectively. The results reveal a convenient and effective method to control the morphology of nonfullerene acceptors and improve the photovoltaic performance of organic solar cells, paving the way for more efficient and practical organic photovoltaic technologies.

## Graphical abstract

A novel strategy for modulating the morphology of BTP‐based acceptors is presented by introducing the low‐cost polar molecule, benzoic anhydride (BA), in combination with 1‐chloronaphthalene. Films of the BTP derivatives processed with the dual additives form a dense fibrous structure, facilitating ordered lamellar packing and *π*–*π* stacking, enhancing photon response and charge transport. The optimum binary organic solar cells exhibit a remarkable power conversion efficiency of 19.59%.

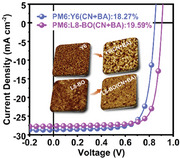

## 1 Introduction

Nonfullerene acceptors (NFAs), valued for their narrow bandgap, strong near‐infrared absorption, and low energy disorder, have driven significant enhancements in the power conversion efficiency (PCE) of organic solar cells (OSCs), achieving PCEs over 20% in both single‐junction and tandem configurations.^[^ ^1^, ^2^ ^]^ Extensive research has focused on developing novel NFA materials through molecular design, particularly since the introduction of advanced pseudo‐2D NFAs such as BTP‐F (Y6).^[^ ^3^, ^4^, ^5^ ^]^ Apart from the molecular structure, the aggregation behavior of NFAs is also crucial for device performance; refining the active layer morphology enhances intermolecular *π*–*π* interactions, facilitating exciton dissociation and charge transport.^[^ ^2^, ^6^, ^7^, ^8^ ^]^

Although the efficiency of OSCs comprising polymer: NFAs blends have significantly improved, particularly with Y6 derivatives, the quantum efficiency of hole transfer from the excited NFAs to the polymer donor remains limited. This limitation results in a lower external quantum efficiency (EQE) response in the acceptor region than that of the donor component.^[^ ^9^, ^10^ ^]^ Small molecule NFAs typically have shorter conjugated lengths, and the efficiency of charge transfer between NFAs largely depends on their conjugation and aggregation behaviors.^[^ ^11^, ^12^, ^13^, ^14^ ^]^ Therefore, controlling the aggregation of NFAs is essential for enhancing charge transfer efficiency.^[^ ^15^ ^]^ The additive‐induced aggregation of NFAs has been demonstrated to significantly enhance the short‐circuit current density (J_SC_) and fill factor (FF) of OSCs, leading to improved PCE.^[^ ^16^, ^17^, ^18^, ^19^ ^]^ The introduction of solid additives such as 1,3‐dibromo‐5‐chlorobenzene, 3,5‐dichlorobromobenzene, and 1,3,5‐tribromobenzene, as well as liquid additives like 1‐chloronaphthalene and 1‐fluoronaphthalene, promotes the NFA aggregation and effectively improves the crystallinity of the active layer.^[^ ^20^, ^21^, ^22^, ^23^, ^24^, ^25^, ^26^ ^]^ For example, morphology regulation using the solid additive 1,3,5‐trimethoxybenzene (TMB) helps to restrict the energetic disorder. Using TMB controls the crystallization of NFAs, thereby significantly enhancing the acceptor photon response in the 700–850 nm range, achieving an EQE value of over 86%.^[^ ^27^ ^]^ Combining the solvent additive 1‐fluoronaphthalene (FN) with different solvents facilitated the formation of a continuous and fine fibril network in L8‐BO. The dense and compact L8‐BO fibrils resulted in enhanced light absorption and charge transport, leading to a superior PCE of 19.0%.^[^ ^28^ ^]^ Recently, polymorphs with varied orientations and scales have been formed in neat Y6 films cast from the poor solvent dichloromethane (DCM) with three high‐boiling‐point additives: FN, 1‐chloronaphthalene (CN), and 1‐bromonaphthalene (BN). The optimized acceptor exhibits a strong photon‐to‐electron response in the 600–800 nm range, significantly enhancing the photocurrent.^[^ ^29^ ^]^ Optimizing the acceptor morphology is an essential strategy for improving the performance of OSCs. While single additives have been widely used, multistep regulation has shown greater potential in enhancing device performance. For instance, the joint introduction of C_60_‐/C_70_‐PCBM and C_60_‐/C_70_‐ICBA fullerene materials optimizes the morphology of PDI‐series acceptors, leading to improved crystallization and higher J_SC_, open circuit voltage (V_OC_), and FF.^[^ ^30^ ^]^ We speculate that adopting multistep strategies can overcome the limitations associated with single additives, thereby achieving more balanced and efficient charge transfer in OSCs.

In this contribution, we introduce a novel, low‐cost polar molecule, benzoic anhydride (BA), as a solid additive to modulate the active layer morphology in combination with the liquid solvent additive CN. BA has a symmetric structure featuring one anhydride group and two benzene rings, which generates an electric dipole moment due to its uneven electronegativity. Using BA and CN effectively regulates the morphology of a series of NFAs, such as Y6, BTP‐eC9, L8‐BO, and N3, enhancing thin film crystallinity, expanding aggregation size, and suppressing energy disorders. Notably, the dual additives induce the self‐assembly of L8‐BO into fine fibers with dense polymorph structures, significantly enhancing the acceptor photon response in the 700–850 nm range and achieving a high EQE value exceeding 80%. As a result, the OSCs composed of PM6:Y6 and PM6:L8‐BO achieve an excellent PCE of 18.27% and 19.59%, respectively. The introduction of BA as an additive holds significant potential for advancing the field by offering a cost‐effective approach to morphology control, potentially leading to more widespread adoption of high‐efficiency OSCs.

## 2 Results and Discussion

**Figure 1:**
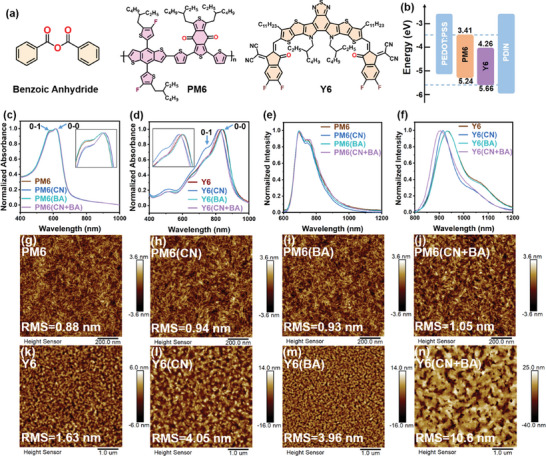
a) Chemical structures of Benzoic Anhydride, PM6, and Y6. b) Energy level diagrams of PM6 and Y6. c,d) Normalized UV–vis absorbance spectra of PM6 and Y6 films processes with and without additives. e,f) Normalized photoluminescence spectra of PM6 and Y6 films processes with and without additives. AFM topography of g–j) PM6 and k–n) Y6 films processes with and without additives.

The chemical structures and energy levels of the materials used in this work are shown in **Figures** **1**a,b and S1 (Supporting Information), respectively. The energy levels between PM6 and Y6 are suitably aligned, as illustrated in Figure 1b, with the lowest unoccupied molecular orbital (LUMO) energy level offset of 0.85 eV and the highest occupied molecular orbital (HOMO) energy level offset of 0.42 eV, providing a strong driving force for exciton dissociation. UV photoelectron spectra are presented in Figure S2 (panels a‐h, Supporting Information), and the values of the different energy levels are summarized in Table S1 (Supporting Information).

To validate the volatility properties of BA, we performed Fourier transform infrared (FTIR) spectroscopy, as depicted in Figure S3 (Supporting Information). Notably, the distinctive peaks of 1211, 1595, and 3061 cm^−1^ to the functional groups of BA were no longer detectable, thereby confirming the complete evaporation of BA from the films subsequent to the annealing process. Furthermore, the efficacy of additive removal is further evidenced by Raman spectroscopy (Figure S4, Supporting Information). The miscibility of the photoactive materials with the additives was evaluated using the Flory‐Huggins interaction parameter derived from thin film surface energy. The results are presented in Figure S5 and Table S2 (Supporting Information). According to the formula $\chi_{donor-accepter}=K{\left(\sqrt{\gamma_{donor}}-\sqrt{\gamma_{accepter}}\right)}^{2}$, the calculated χ values are 0.52, 0.002, 0.84, and 0.04 K mJ m^−2^ for PM6:Y6, PM6:Y6(BA), PM6(BA): Y6, and PM6(BA): Y6(BA), respectively, which suggests that the BA additives exhibit better miscibility with Y6 than with PM6. We calculated the interactions between PM6‐BA and Y6‐BA (Figures S6–S8, Supporting Information). The optimized geometry and IRI map show the stacking distance of BA‐Y6 (3.4 Å) less than the stacking distance of BA‐PM6 (3.5 Å), suggesting strong interactions between BA and the Y6.

To evaluate the interaction between BA and Y6, the electrostatic potential (ESP) distribution of both molecules was calculated using density functional theory (DFT). As depicted in Figure S9 (Supporting Information), the ESP distribution, guided by the principle of opposite polarity attraction, suggests robust intermolecular interactions between BA and the core units of BTP. Next, we calculated the adsorption energy (E_ad_) using molecular dynamics (MD) simulations. Single crystal analysis revealed three distinct packing models for BTP‐series molecules: S1, S2, and W. We therefore simulated the adsorption of BA onto each configuration. Figures S10–S12 (Supporting Information) show that BA exhibits the most negative adsorption energy (E_ad_: −1.10 eV) when adsorbed to the S2 configuration, compared to −0.87 eV for S1 and −0.78 eV for W. This indicates that the S2 configuration allows for tighter molecular packing under the influence of BA. As shown in Figures S13 and S14 (Supporting Information), MD simulation results reveal that the distance between Y6 molecules is ≈3.63 Å. Upon adsorption of the BA molecule, this distance decreases to 3.59 Å, underscoring the strong regulatory effect of the rigid BA molecule on Y6's arrangement. Based on these simulation findings, we conducted experiments to validate the results. The experimental data corroborate the simulations, confirming the significant role of BA in enhancing molecular ordering and intermolecular interactions within the BTP‐series molecules.

The absorbance spectra of neat PM6, Y6, and BA films are presented in Figure S15a (Supporting Information). Figure S15b (Supporting Information) shows the normalized absorbance spectra of PM6:Y6 films with and without additives. While the blend of PM6:Y6 covers the wavelength range from 300 to 900 nm, BA exhibits an obvious absorption peak in the range of 200–350 nm. The impact of the additives on the thin‐film optical properties was analyzed. Figure 1c shows the normalized absorbance spectra of the following films: PM6, PM6 processed with CN (PM6:CN), PM6 processed with BA (PM6:BA), and PM6 processed with both CN and BA (PM6:CN+BA). All films exhibit two characteristic transition spectral peaks labeled 0‐0 and 0–1. As depicted in the enlarged detail within the figure, the spectral peaks of the PM6:CN and PM6:CN+BA films shift toward longer wavelengths relative to the control film (PM6). Concurrently, the intensity ratio of the 0–1 to 0‐0 transition peaks is notably reduced compared to the control film. In contrast, the spectral peaks of the PM6:BA and PM6:CN+BA films do not show an apparent shift compared to the PM6 and PM6:CN films, respectively. However, the 0–1/0‐0 intensity ratio for the PM6:BA and PM6:CN+BA films is slightly higher than that of the PM6 and PM6:CN films. Considering the spectral shifts and changes in peak intensity ratios, the introduction of CN causes J‐type aggregation of PM6, while using BA alone results in less ordered or H‐type aggregation of PM6.^[^ ^29^, ^31^, ^32^ ^]^

Using additives significantly affects the absorbance spectra of Y6, as depicted in Figure 1d. The spectra fitting details are provided in Figure S16 (Supporting Information). A unique vibronic pattern is observed in the spectra of Y6 with and without additives, consisting of four distinct components: A_0‐0_, A_0‐1_, A_0‐2_, and A_0‐3_. The ratio of A_0‐0_/A_0‐1_ is often used to analyze the molecular aggregation characteristics, while the ratio of full width at half maximum (FWHM_0‐0_/FWHM_0‐1_) is used to evaluate the order of thin films.^[^ ^29^ ^]^ The parameters derived from the absorbance spectra of the Y6 thin film are listed in Table S3 (Supporting Information). Compared to neat Y6 thin film, the A_0‐0_/A_0‐1_ value of Y6 film processed with CN (Y6:CN) significantly increased to 3.13, indicating enhanced J‐type aggregation. The A_0‐0_/A_0‐1_ value of Y6 film cast with BA (Y6: BA) increased to 2.48, suggesting slightly enhanced extent of J‐type aggregation in the films. When CN and BA are simultaneously applied to process Y6 film (Y6:CN+BA), the A_0‐0_/A_0‐1_ value is 2.80, which lies between 2.48 and 3.13 but is still greater than the neat Y6 film, indicating that the synergistic effect of CN and BA still enhances the J‐aggregation of Y6 film. The Y6:CN film exhibits a larger FWHM_0‐0_/FWHM_0‐1_ ratio compared to the neat Y6 film, while the Y6:BA film shows a lower FWHM_0‐0_/FWHM_0‐1_ ratio, indicating that CN and BA have opposite effects on the order of the Y6. Additionally, the 0‐0 peaks of the Y6:CN and Y6:BA films exhibit a blueshift of 17 nm and a redshift of 6 nm, respectively, further confirming that CN and BA have opposite effects on the order of the Y6 film. Thus, using BA in combination with CN, rather than CN alone, effectively limits the large extent of aggregation induced by CN, contributing to a finely tuned active layer morphology. Besides the noticeable variations in the absorbance spectra, the additives also alter the photoluminescence (PL) spectra. The steady‐state PL spectra in Figure 1e,f and the intensity spectra in Figure S17a,b (Supporting Information) indicate that the synergistic effect of CN+BA has corresponding impacts on both the donor and acceptor, consistent with the absorbance analysis. Nonetheless, the effect of the dual additive on the acceptor is greater than that on the donor. The Stokes shift (ΔE_S_) is also evaluated using the absorbance and PL spectra (Figure S18, Supporting Information). Compared to neat Y6 films, Y6 films processed with CN and BA have a reduced ΔE_S_. A finite energy disorder alleviates the vibrational relaxation, resulting in a limited ΔE_S_. Therefore, CN and BA synergistically lead to an ordered stacking of Y6 and suppress the energy disorder.^[^ ^24^ ^]^

Changes in the nanoscale morphology directly influence the optical properties of the thin films.^[^ ^29^, ^33^ ^]^ To investigate the effect of additives on the morphology of donors and acceptors, we monitored the topography of the donor and acceptor films using atomic force microscopy (AFM). First, as shown in Figure 1g–j, the root‐mean‐square roughness (RMS) of both PM6:CN and PM6:BA films is 0.94 and 0.93 nm, respectively, which is increased compared to the neat PM6 film (0.88 nm), indicating slightly increased crystallinity of PM6 processed with additives. When CN and BA are introduced into PM6 simultaneously, the RMS is the highest among the films, suggesting that the synergistic effect of the dual additives further promotes the crystallinity of PM6. Second, as shown in Figure 1k–n, the RMS values of the Y6:CN and Y6:BA films are 4.05 and 3.96 nm, respectively, which are higher than the roughness of the neat Y6 film (1.63 nm), indicating that both additives enhance the crystallinity of Y6. When both additives are applied to Y6 simultaneously, the RMS increases to 10.6 nm, more than twice that of the other films. A significant change in the Y6: CN+BA film morphology is also observed. The dramatic morphology changes in the Y6:CN+BA film show that the dual additives effectively modulate the microstructure of the acceptor layer, further proving the synergistic effect on the acceptor.

The crystalline features of the Y6 films processed with and without additives were measured using grazing incident wide‐angle X‐ray scattering (GIWAXS), and the 2D GIWAXS patterns are shown in Figure S19 (Supporting Information). The lamella stacking distance (*d*‐spacing) and crystalline coherence length (CCL) were calculated using the Scherrer equation, and the results are summarized in Table S4 (Supporting Information). The changes in *d*‐spacing indicate that the CN and BA additives are beneficial for Y6 to achieve a more ordered lamellar packing and π–π stacking. When CN and BA are simultaneously applied to Y6, the *d*‐spacing value in the in‐plane (IP) (110) falls between those of Y6:CN and Y6:BA, while the *d*‐spacing value is minimized to 3.51 in the out‐of‐plane (OOP) (010). This suggests that the synergistic effect of CN and BA achieves a smaller *π*–*π* stacking distance in Y6 films, thereby reducing the steric hindrance between molecules in the OOP direction,^[^ ^19^ ^]^ which is conducive to charge transfer between molecules. Further evidence supporting the promotion of carrier transport comes from the enhanced crystallinity observed in both the IP (110) and OOP (010) directions, as implied by the changes in CCL.^[^ ^21^, ^34^ ^]^

The crystallization and aggregation behavior during the spin‐coating processes for Y6 thin film systems were examined using in situ absorption measurements (interval of 0.02 s per data point). Figure S20a–h (Supporting Information) display the contour images of spin‐coating processes, and three stages (I:< 0.1 s, II:0.1–0.3 s, III:> 0.3 s) are observed during spin‐coating. Due to their high crystallinity, the absorption peaks of Y6, Y6:CN, Y6:BA, and Y6:CN+BA exhibit significant red‐shifted during the film‐formation process. Their film‐forming times are 0.177, 0.198, 0.196, and 0.238 s, respectively, suggesting that Y6:CN+BA film has a longer crystallization time. The extended crystallization period of Y6:CN+BA film provides sufficient time for crystal growth, leading to more ordered molecular stacking and high crystallinity. Among these stages, stage II is a pivotal process for film formation as it determines the crystallization kinetics of the film and, hence, the crystallinity of the film. During this stage, the peak location of the Y6 film sharply increased, and the 0‐0 intensity transitioned rapidly within 0.1–0.3 s. The peak location of the Y6:CN and Y6:BA film slowly increased, yet the emergence of other peaks accompanied the 0‐0 peak intensity of the Y6:CN film. The variation in the 0‐0 peak intensity of the Y6:BA film is very pronounced and distinct. When BA and CN are simultaneously applied to Y6, it can be observed that there is a more gradual increase in the peak position of the film. In contrast, the 0‐0 peak intensity variation is discernible, which indicates that Y6:CN+BA film has more ordered molecular stacking and high crystallinity.

**Figure 2:**
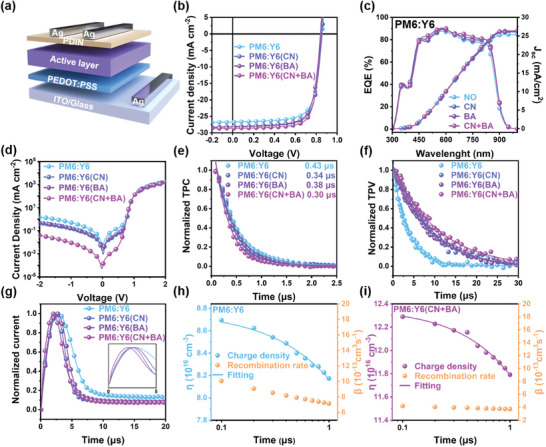
a) The scheme of device configuration. b) *J*–*V* curves of the optimal PM6:Y6, PM6:Y6(CN), PM6:Y6(BA), and PM6:Y6(CN+BA) devices under the illumination of AM 1.5G irradiance (100 mW cm^−2^). c) EQE spectra and d) dark *J*–*V* characteristics of devices based on PM6:Y6 with and without additives. e) Normalized photocurrent decay (TPC), f) photovoltage decay (TPV), and g) Photo CELIV current transients of of devices based on PM6:Y6 with and without additives. Charge carrier density of h) PM6:Y6 and i) PM6:Y6(CN+BA) devices as a function of delay time and the bimolecular recombination fits (solid lines).

We fabricated OSCs with a device structure of ITO/PEDOT:PSS/active layer (120 nm)/PDIN/Ag to evaluate the impact of dual additive regulation of the acceptor phase on the photovoltaic performance, as plotted in **Figure** **2**a. Subsequently, we conducted an optimization process for additive concentrations of BA and CN additives, as indicated in Figure S21a,b and summarized in Table S5 (Supporting Information).The current density voltage (*J*–*V*) curves of OSCs were measured with simulated AM 1.5G irradiation (100 mW cm^−2^). The detailed device parameters are summarized in **Table** **1**. The control devices (PM6:Y6) yield a V_OC_ of 0.86 V, a J_SC_ of 26.7 mA cm^−2^, an FF of 72.45%, and a PCE of 16.57%, consistent with previous reports.^[^ ^21^, ^23^, ^33^ ^]^ Devices processed with CN achieved a maximum PCE of 17.73%, with a V_OC_ of 0.85 V, a J_SC_ of 28.24 mA cm^−^^2^, and an FF of 73.52%. BA‐treated devices had a maximum PCE of 17.33%, a V_OC_ of 0.84 V, a J_SC_ of 28.09 mA cm^−^^2^, and an FF of 73.39%. Compared to the control device, these efficiencies represent a significant improvement. For the devices with both CN and BA, the photovoltaic parameters were significantly improved, attaining a V_OC_ of 0.85 V, a J_SC_ of 28.61 mA cm^−2^, and an FF of 75.49%, resulting in an optimum PCE of 18.27%. The apparent increase in the device performance suggests that the dual additive regulation of acceptor morphology is a practical approach. The EQE spectra of the corresponding devices are illustrated in Figure 2c, exhibiting high EQE values in the wavelength range of 300–900 nm. Notably, after 700 nm, a significant change in the EQE value of the acceptor is observed. Compared to the control devices, the devices with CN and BA exhibit a considerable improvement. This trend aligns with the device's current density increase, indicating that the higher current density primarily stems from the acceptor's contribution.^[^ ^27^, ^29^ ^]^ Besides optimizing the photocurrent, the dark is also improved. The devices processed with CN+BA exhibit the lowest current leakage (Figure 2d), demonstrating the positive impact of the dual additives. Current leakage measures charge recombination and extraction efficiency in the device.^[^ ^34^, ^35^ ^]^ Thus, the suppressed current leakage in the devices processed with dual additives is consistent with the higher FF and J_SC_ obtained.

**Table 1:** The photovoltaic parameters of the optimized OSCs based on PM6:Y6 were measured under AM 1.5G (100 mW cm^−2^) simulated irradiance.

Photoactive layer	V_OC_[V]	J_SC_[mA cm^−2^]	FF[%]	PCE[%]^a)^
PM6:Y6	0.86 (0.85 ± 0.005)	26.70 (27.00 ± 0.43)	72.45 (71.32 ± 0.91)	16.57 (16.41 ± 0.12)
PM6:Y6(CN)	0.85 (0.85 ± 0.001)	28.24 (28.00 ± 0.23)	73.52 (72.99 ± 1.02)	17.73 (17.47 ± 0.24)
PM6:Y6(BA)	0.84 (0.85 ± 0.009)	28.09 (27.64 ± 0.32)	73.39 (73.08 ± 0.82)	17.33 (17.16 ± 0.16)
PM6:Y6(CN+BA)	0.85 (0.85 ± 0.002)	28.61 (28.47 ± 0.39)	75.49 (75.33 ± 0.87)	18.27 (18.16 ± 0.14)

^a)^ The statistics were obtained from 10 devices for each condition.

Next, we performed time‐resolved optoelectronic measurements to understand carrier dynamics, including charge extraction, charge carrier lifetimes, and recombination properties.^[^ ^35^ ^]^ The charge extraction time, determined by transient photocurrent (TPC) measurements, was measured under short‐circuit conditions. The current decay is fitted with a monoexponential decay model and plotted in Figure 2e. The charge extraction times were 0.43, 0.34, 0.38, and 0.30 µs for the PM6:Y6, PM6:Y6(CN), PM6:Y6(BA), and PM6:Y6(CN+BA) devices, respectively. These results imply that the generated charges in the optimized devices are more efficiently extracted before charge recombination occurs.^[^ ^20^, ^21^, ^35^ ^]^ Meanwhile, when the device is held in an open‐circuit voltage condition, the transient photovoltage (TPV) decay provides information about the carrier lifetime of the device. As illustrated in Figure 2f, the lifetimes of the PM6:Y6, PM6:Y6(CN), PM6:Y6(BA), and PM6:Y6(CN+BA) devices are 3.51, 9.28, 8.89, and 10.92 µs, respectively. These results suggest a lesser extent of charge recombination in the PM6:Y6(CN+BA) devices. To quantify the recombination rate in the device, we conducted photo‐induced charge extraction with linearly increasing voltage (photo‐CELIV) measurements. As shown in Figure 2g, the normalized current transients of devices with and without additives are presented. By integrating the current signal, the charge carrier density is estimated. The charge carrier density as a function of delay time is plotted in Figure 2h,i and Figure S22a,b (Supporting Information). The charge density follows a dispersive bimolecular charge recombination behavior, and the bimolecular recombination rate can be derived by fitting the data with the following equation:^[^ ^21^, ^33^, ^35^ ^]^ $\;\beta \left(t\right)=\left(1/\tau_{b}\right)\;\gamma n_{0}^{-1}{\left(t/\tau_{b}\right)}^{\gamma -1}$ where *n*_0_ is the initial charge density, τ_b_ is the recombination time constant, and β(*t*) is the bimolecular recombination rate. The bimolecular recombination rate β(*t*) of PM6:Y6, PM6:Y6(CN), PM6:Y6(BA), and PM6:Y6(CN+BA) devices are 1.01  ×  10^−12^, 4.64  ×  10^−13^, 6.35  ×  10^−13^, and 4.22  ×  10^−13^ cm^−3^ s^−1^, respectively, consistent with the charge carrier lifetime determined by TPV. We examined the trap density of the devices with additives using deep‐level transient spectroscopy (Figure S23, Supporting Information). The results indicate that the introduction of additives decreases the trap density in the devices from 2.23 × 10^16^ to 1.90 × 10^16^ cm^−3^(CN),1.85 × 10^16^ cm^−3^(BA), and 1.68 × 10^16^ cm^−3^(CN+BA). As a result, the reduction in trap density suppresses trap‐assisted recombination, thereby enhancing the FF. The hole (µ_h_) and electron mobility (µ_e_) were fitted using the space charge limiting current model, as shown in Figure S24a,b (Supporting Information). The µ_h_ of PM6:Y6, PM6:Y6(CN), PM6:Y6(BA), and PM6:Y6(CN+BA) devices are 4.5 × 10^−4^, 6.1 × 10^−4^, 5.6 × 10^−4^, and 9.3 × 10^−4 ^cm^2^V^−1^s^−1^, respectively. The µ_e_ of the same devices are 2.6 × 10^−4^, 3.1 × 10^−4^, 2.9 × 10^−4^, and 4.2 × 10^−4^ cm^2^V^−1^s^−1^, respectively. The approach of using dual additives to regulate acceptor morphology significantly improves the FF and J_SC_ of the OSCs, primarily attributed to higher carrier mobility, reduced bimolecular recombination, and longer carrier lifetime.^[^ ^17^, ^30^ ^]^ The long‐term stability of the device is essential. To assess this critical point, we monitored the performance of the devices over the storage period, as shown in Figure S25 (Supporting Information). After 480 h, the additive‐ and control devices show relative stability in V_OC_ and J_SC_, maintaining ≈98% of their initial values. However, the FF of the control device reduces to ≈87% of its initial value, while the PM6:Y6(CN), PM6:Y6(BA), and PM6:Y6(CN+BA) devices retain 89%, 92%, and 94% of their initial FF, respectively. As a result, the PCE of the PM6:Y6(CN+BA) device is preserved at 92%, compared to a decline of 78% for the control device.

**Figure 3:**
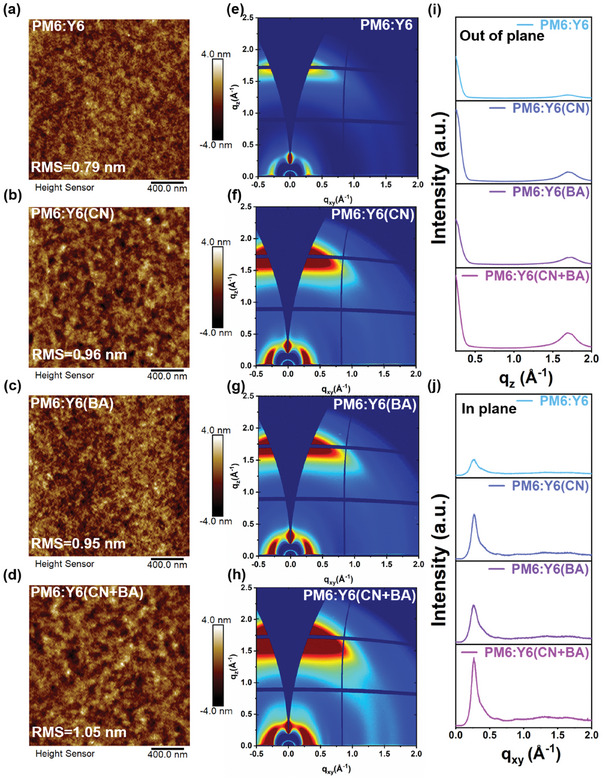
AFM and 2D GIWAXS patterns of the PM6:Y6 blend films processed with and without solid additives. a–d) AFM images of the PM6:Y6, PM6:Y6(CN), PM6:Y6(BA), and PM6:Y6(CN+BA) films. e–h) 2D GIWAXS images of the PM6:Y6, PM6:Y6(CN), PM6:Y6(BA), and PM6:Y6(CN+BA) films. i,j) The out‐of‐plane and in‐plane line cut of the 2D GIWAXS data.

The device performance is directly affected by the active layer morphology. To probe the microstructural changes of the blend films, we conducted atomic force microscopy (AFM) measurements to visualize the morphological changes of the BHJ films. As shown in **Figure** **3**a–d, the surface roughness (RMS) of the neat PM6: Y6 film is ≈0.79 nm. In contrast, the CN‐induced and BA‐induced BHJ films display RMS values of 0.94 and 0.93 nm, respectively, whereas the film processed with CN and BA additives has an RMS value of 1.05 nm. This slight increase in RMS suggests enhanced crystallinity within the thin film.^[^ ^29^ ^]^ The observed trend in RMS values aligns with the morphological changes in the acceptor phase induced by the dual additive treatment. To quantify the enhanced crystalline characteristics of the thin films, we performed GIWAXS measurements to visualize the crystallinity and molecular orientation properties of the BHJ films. As shown in Figure 3e–h, the scattering intensities of the PM6:Y6 films processed with CN+BA are notably stronger compared to the control films, suggesting a more organized molecular aggregation structure.^[36,37]^ The in‐plane (IP) (110), (11‐1), and out‐of‐plane (OOP) (010) peaks from the line cuts are depicted in Figure 3i,j, with their corresponding fitted coherence length (CCL) and *d*‐spacing values provided in Table S6 (Supporting Information). When *q*_xy_ is in the range of 0–0.5 Å−¹, there is a marked increase in the intensity of the IP (110) and (11‐1) peak, following the order: PM6:Y6(CN+BA) > PM6:Y6(CN) > PM6:Y6(BA) > PM6:Y6. Similarly, when *q*_z_ is between 1.5 and 2.0 Å−¹, the OOP (010) peak intensity follows the same trend as the IP (110) and (11‐1) peak, indicating that the synergistic effect of CN and BA additives enhances the crystallinity of the active layer film in both IP and OOP directions. Combined with the fitted data, the trend in CCL change is entirely consistent with the peak intensity changes. For the IP direction, the CCL values of PM6:Y6(CN+BA), PM6:Y6(CN), PM6:Y6(BA), and PM6:Y6 are 72.50, 69.81, 58.90, and 57.70 Å, respectively, with corresponding *d*‐spacing values of 26.80, 25.13, 24.91, and 23.76 Å. For the OOP direction, the CCL values of PM6:Y6(CN+BA), PM6:Y6(CN), PM6:Y6(BA), and PM6:Y6 are 28.56, 28.27, 26.92, and 26.42 Å, respectively. Meanwhile, we analyzed the line cuts along specific scattering rings to investigate intensity variation as a function of polar angle (χ), as shown in Figure S26 (Supporting Information). The intensity variations in the (010) and (110) directions are consistent with the changes in CCL. The GIWAXS analysis demonstrates that the use of dual additives enhances the crystallinity of the active layer, thereby promoting the carrier transport properties. Similarly, we performed in situ UV–vis absorption measurements to evaluate the blend film‐formation dynamics. Figure S27 (Supporting Information) depicts that the donor's film‐forming process did not exhibit significant changes in the blend film. However, the film‐forming process of the acceptor in the blend film was highly consistent with that of the neat acceptor film, which implies that the additive primarily modulates molecular stacking and crystallinity of the film by influencing the acceptor in the blend film. This also fully demonstrates the feasibility of modulating the acceptor phase through a dual‐additive approach.

**Figure 4:**
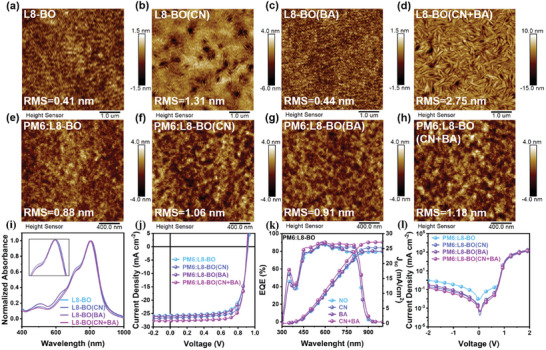
AFM topography of a–d) L8‐BO and e–h) PM6:L8‐BO films processes with and without additives. i) Normalized UV–vis absorption spectra of L8‐BO films processes with and without additives. j) *J*–*V* curves of the optimal PM6:L8‐BO, PM6:L8‐BO(CN), PM6:L8‐BO(BA), and PM6:L8‐BO(CN+BA) devices under the illumination of AM 1.5G irradiance (100 mW cm^−2^). k) EQE spectra and l) dark *J*–*V* characteristics of devices based on PM6:L8‐BO with and without additives.

We detailly analyzed the impact of dual additives on the device performance and morphological changes. Beyond the influence on Y6, we applied this method to Y6 derivatives, including L8‐BO, BTP‐eC9, and N3. The morphological variations and the device performance were systematically recorded. To further confirm the synergistic effect of CN and BA on the acceptor morphology, AFM images of L8‐BO, BTP‐eC9, and N3 films were taken separately, as shown in **Figures** **4**a–d and S28a–h (Supporting Information). The dual additives exert distinct effects on the morphology of different acceptors, leading to improved roughness and enhanced crystallinity. Notably, when CN and BA are used together, the morphology changes significantly, particularly in L8‐BO, where the formation of rod‐shaped fibers is distinctly observed in Figure 4d, indicating that the dual additive strategy can effectively regulate the morphology of various acceptors. The changes in optical properties of L8‐BO, BTP‐eC9, and N3 due to morphological modifications are evident from the normalized absorbance spectra presented in Figure 4i and Figure S28i–j (Supporting Information), respectively. These results support the effectiveness of using CN and BA additives in tailoring the acceptor morphology, thereby improving OSC performance.

As an example, the *J*–*V*, EQE, and dark *J*–*V* characteristics of devices composed of PM6:L8‐BO under different processing conditions are shown in Figure 4j–l, respectively. The detailed *J*–*V* parameters are summarized in **Table** **2**. Notably, the OSCs processed with CN and BA additives exhibited an optimal PCE of 19.59%, with an average efficiency of 19.26%. This performance is associated with a V_OC_ of 0.91 V, a J_SC_ of 27.70 mA cm^−^^2^, and an FF of 78.14%, surpassing that of devices processed with CN or BA alone. The EQE spectrum of PM6:L8‐BO (CN+BA) shows a significant enhancement in the range of 700–850 nm, indicating that the synergistic effect of CN and BA additives enhances the photon‐to‐electron conversion of the acceptor material,^[^ ^27^ ^]^ consistent with the findings in PM6:Y6 (CN+BA) systems. The dark *J*–*V* characteristics indicate low leakage currents in the PM6:L8‐BO (CN+BA) device. This correlates well with the higher FF achieved, suggesting reduced non‐radiative recombination losses and improved charge collection efficiency.^[^ ^21^ ^]^

**Table 2:** The photovoltaic parameters of the optimized OSCs based on PM6:L8‐BO were measured under AM 1.5G (100 mW cm^−2^) simulated irradiance.

Photoactive layer	V_OC_ [V]	J_SC_[mA cm^−2^]	FF[%]	PCE[%]^a)^
PM6:L8‐BO	0.92 (0.92 ± 0.001)	25.72 (25.73 ± 0.18)	71.90 (71.80 ± 0.26)	17.01 (16.99 ± 0.08)
PM6:L8‐BO(CN)	0.91 (0.91 ± 0.004)	26.58 (26.35 ± 0.21)	76.13 (76.21 ± 0.42)	18.45 (18.19 ± 0.21)
PM6:L8‐BO(BA)	0.92 (0.92 ± 0.001)	25.96 (25.81 ± 0.11)	73.10 (72.94 ± 0.33)	17.39 (17.23 ± 0.09)
PM6:L8‐BO(CN+BA)	0.91 (0.90 ± 0.005)	27.70 (27.60 ± 0.29)	78.14 (77.82 ± 0.38)	19.59 (19.26 ± 0.20)

^a)^ The statistics were obtained from 10 devices for each condition.

Additionally, the *J*–*V* characteristics and detailed parameters for PM6:BTP‐eC9 and PM6:N3 under different processing conditions are illustrated in Figure S18k,l (Supporting Information) and summarized in Tables S7 and S8 (Supporting Information). The introduction of CN and BA elevates the PCE from 17.47% to 18.59% for PM6:BTP‐eC9 and from 15.64% to 18.52% for PM6:N3. The synergistic impact of CN and BA additives finely tunes the morphology of the acceptor phases, leading to improvements in the photovoltaic performance of OSCs. This underscores the broad applicability of this dual additive strategy across different acceptor materials, demonstrating its potential for enhancing device efficiencies.

## 3 Conclusion

In summary, we modulated the aggregation of BTP‐based NFAs using a dual additive strategy, promoting the formation of fine fibers with dense molecular structures in BTP‐based derivatives. Altering the acceptor morphology facilitated more ordered lamellar packing and *π*–*π* stacking, leading to favorable phase separation, enhanced molecular stacking, and improved crystallinity, which resulted in higher charge mobility. Optimized acceptor morphology promoted exciton diffusion and segregation, reduced charge extraction time, improved carrier lifetime, accelerated charge transport, and suppressed charge recombination, thereby enhancing the photovoltaic performance of OSCs with PM6:Y6, PM6:L8‐BO, PM6:BTP‐eC9, and PM6:N3. Specifically, devices composed of PM6:Y6 processed with dual additives achieved a PCE of 18.27%, while PM6:L8‐BO reached a PCE of 19.59%. These results highlight the effectiveness of controlling the morphology of NFAs to improve the performance of OSCs, providing valuable insights for developing more efficient and practical processing additives.

## Conflict of Interest

The authors declare no conflict of interest.

## Supporting information



Supporting Information

## Data Availability

Research data are not shared.
