# Supplementary material for: Modulating Acceptor Phase Leads to 19.59% Efficiency Organic Solar Cells
Source: Adv Sci (Weinh). 2024 Dec 24;12(7):2413051. doi: 10.1002/advs.202413051 (PMC11831495; doi:10.1002/advs.202413051)
Supplement: Supplementary file 1 — Supporting Information [file ADVS-12-2413051-s001.docx]

Supporting Information

**Modulating Acceptor Phase Leads to 19.59% Efficiency Organic Solar Cells**

Liang Bai,^1^ Sein Chung,^2^ Zhenmin Zhao,^1^ Jingjing Zhao,^1^ Yuqing Sun,^1^ Yuan Liu,^1^ Lixing Tan,^1^ Jiancheng Zhong, ^1^ Sooji Lyu,^2^ Hojun Ji,^2^ Kilwon Cho,^2^ and Zhipeng Kan*^1,3^

^1^L. Bai, Z. Zhao, J. Zhao, Y. Sun, Y. Liu, L. Tan, J. Zhong, Z. Kan

Center on Nanoenergy Research, Carbon Peak and Neutrality Science and Technology Development Institute, School of Physical Science & Technology, Guangxi University, Nanning 530004, China.
^2^S. Chung, S. Lyu, H. Ji, K. Cho

Department of Chemical Engineering, Pohang University of Science and Technology, Pohang 37673, South Korea.

^3^Z. Kan

State Key Laboratory of Featured Metal Materials and Life-cycle Safety for Composite Structures, Nanning 530004, China

Corresponding Author

Zhipeng Kan - Center on Nanoenergy Research, Institute of Science and Technology for Carbon Peak & Neutrality, School of Physical Science & Technology, Guangxi University, Nanning 530004, China.

E-mail: [kanzhipeng@gxu.edu.cn](mailto:kanzhipeng@gxu.edu.cn)

**1. Materials.**

PM6, Y6, BTP-eC9, L8-BO and N3 were purchased from Solarmer Co., Ltd. (Beijing, China). BA was purchased from aladdin (Shanghai, China). PEDOT:PSS and PDIN were purchased from Energy Chemical., Ltd. These materials were used as received without further treatment.

**2. Device fabrication and characterization of the OSCs**

All the devices are based on a conventional sandwich structure, patterned ITO glass/PEDOT:PSS/active layer/PDIN/Ag. The ITO substrates were first scrubbed by detergent and then sonicated with deionized water, acetone and isopropanol subsequently, and dried in an oven. The glass substrates were treated by UV-ozone for 30 min before use. PEDOT:PSS (Al4083 from Hareus) was spin-cast onto the ITO substrates at 5000 rpm for 20 s, and then dried at 150 °C for 20 min, and the substrates were then transferred into the glovebox for active layer deposition. The PM6:Y6 blends (weight ratios are: 1:1.2), were dissolved in chloroform (the concentration of donor was 7 mg mL-1), with BA (1mg/ml) and/or 1-chloronaphthalene (0.5 wt%) as additive(s), and stirred for 3h at 50 degrees. The blend solution was spin-cast at 3500 rpm for 30 s onto PEDOT:PSS-TA film followed by a temperature annealing of 75°C for 5 min. A thin PDIN layer (0.5 mg/mL in methanol and 0.3 vol% acetic, 5000 rpm) was coated on the active layer, followed by the deposition of Ag (evaporated under 3×10^-4^ Pa through a shadow mask). The J−V measurement was performed via a XES-50S1 (SAN-EI Electric Co., Ltd.) solar simulator (AAA grade) whose intensity was calibrated by a certified standard silicon solar cell (SRC-2020, Enlitech) under illumination of AM 1.5G 100 mW cm^-2^. The AM 1.5G light source with a spectral mismatch factor of 1.01 was calibrated by the National Institute of Metrology. The intensity of the AM 1.5G spectra was calibrated by a certified standard silicon solar cell (SRC-2020, Enlitech) calibrated by the National Institute of Metrology. The J-V curves of small-area devices were measured in forwarding scan mode (from -0.2 V to 1.2 V) with a scan step length of 0.02 V. The external quantum efficiency (EQE) was measured by a certified incident photon to electron conversion (IPCE) equipment (QE-R) from Enli Technology Co., Lt. The light intensity at each wavelength was calibrated using a standard monocrystalline Si photovoltaic cell.

**3. TPC, TPV Measurements**

For TPV, the measurement was conducted under 1 sun condition by illuminating the device with a white light-emitting diode, and the champion device is set to the open-circuit condition. For TPC, the champion device is set to the short-circuit condition in dark. The output signal was collected by key sight oscilloscope. The transient photocurrent (TPC) was tested under the short-circuit condition to explore the time-dependent extraction of photogenerated charge carriers. The 10 ns light plus laser were selected as the light source for steady the photogenerated current density. The devices are otherwise kept in the dark between pulsesto avoid any influence of pulse frequency on the current responses. The transient photovoltage (TPV) was tested under the open-circuit condition to explore the photovoltage decay.

**4. photo-CELIV Measurements**

The photo-CELIV measurements were conducted using the all-in-onecharacterization platform Paios, developed and commercialized by Fluxim AG, Switzerland. To ensure accurate results, all devices were prepared for photo-CELIV measurements in accordance with the relevant device fabrication conditions. The ramp rate was set at 0.10 V/us, the delay time was 70 us, the light pulse length was 30 us, and the setup-type was LED.

**5. SCLC Measurements**

The electron and hole mobilities were measured by using the method of space-charge limited current (SCLC) for electron-only devices with the structure of ITO/ZnO/active layer/PNDIT-F3N/Ag and hole-only devices with the structure of ITO/PEDOT:PSS-TA/active layers/MoO_x_/Ag. The charge carrier mobility was determined by fitting the dark current to the model of a single carrier SCLC according to the equation: *J* = 9*ε*_0_*ε*_r_*μV^2^*/8*d^3^*, where *J* is the current density, *d* is the film thickness of the active layer, *μ* is the charge carrier mobility, *ε*_r_ is the relative dielectric constant of the transport medium, and *ε*_0_ is the permittivity of free space. *V* = *V*_app_ –*V*_bi_, where *V*_app_ is the applied voltage, *V*_bi_ is the built-in voltage. The carrier mobility is calculated from the slope of the *J*^1/2^ ~ *V* curves.

**6. GIWAXS analysis**

Grazing-incidence wide-angle X-ray scattering (GIWAXS) was carried out to investigate the molecular packing and molecular orientation in the thin films. The π–π stacking distance (d-spacing) and crystalline coherence length (CCL) were calculated quantitatively using the equations d-spacing = 2π/q and CCL = 2πK/Δq, where q, Δq and the K constant represent the peak positions, full width at half maximum and order unit which takes on dimensions, respectively. The K value is 0.9.

**7. DLTS Measurements**

This experiment, a method of transient photocurrent was used to the DLTS measurement. The carrier emission process is observed as a current transient in the device current signal. By following equation according to experimental data the trapped defect state volume density N_t_ can be given:

$$j_{te}\left( t \right)=\frac{1}{{}_{te}}\cdot q\cdot d\cdot N_{t}\cdot exp(-\frac{t}{{}_{te}})$$

Here, $j_{te}$ is trap emission current, ${}_{te}$ is catch-trap emission time constant, q is a single charge amount, d is the thickness of the device.

**8. Density Functional Theory (DFT) Calculations**

The structural optimization is performed via the Vienna Ab initio Simulation Package (VASP). To obtain the ground state properties, using a projector-augmented wave (PAW) pseudopotential in conjunction with the Perdew–Burke–Ernzerhof (PBE) functional 2 and plane-wave basis set with energy cutoff at 400 eV. The atomic structure of systems are positioned in a cubic supercell of 42×34×21 Å^3^ along three directions and fully relaxed until the force on each atom was less than 0.02 eV/Å. Monkhorst Pack k-point mesh of 1×1×1 was adopted for the calculations.


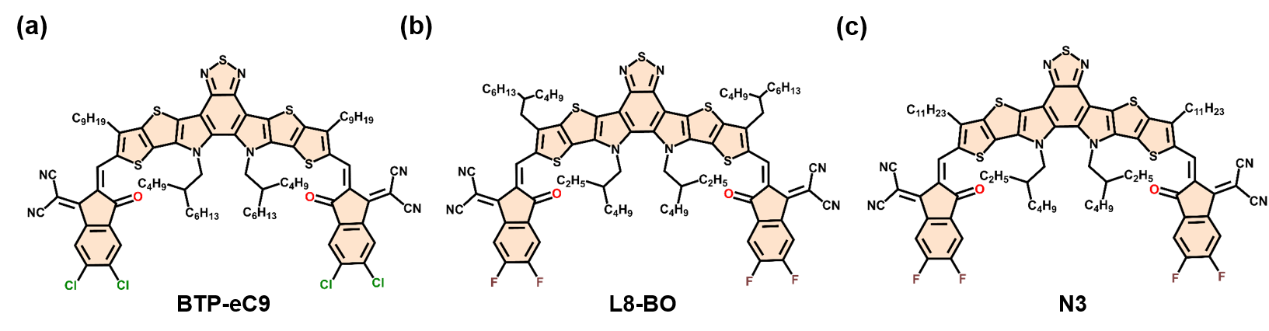


**Figure S1.** Chemical structures of (a) BTP-eC9, (b) L8-BO, and (c) N3.


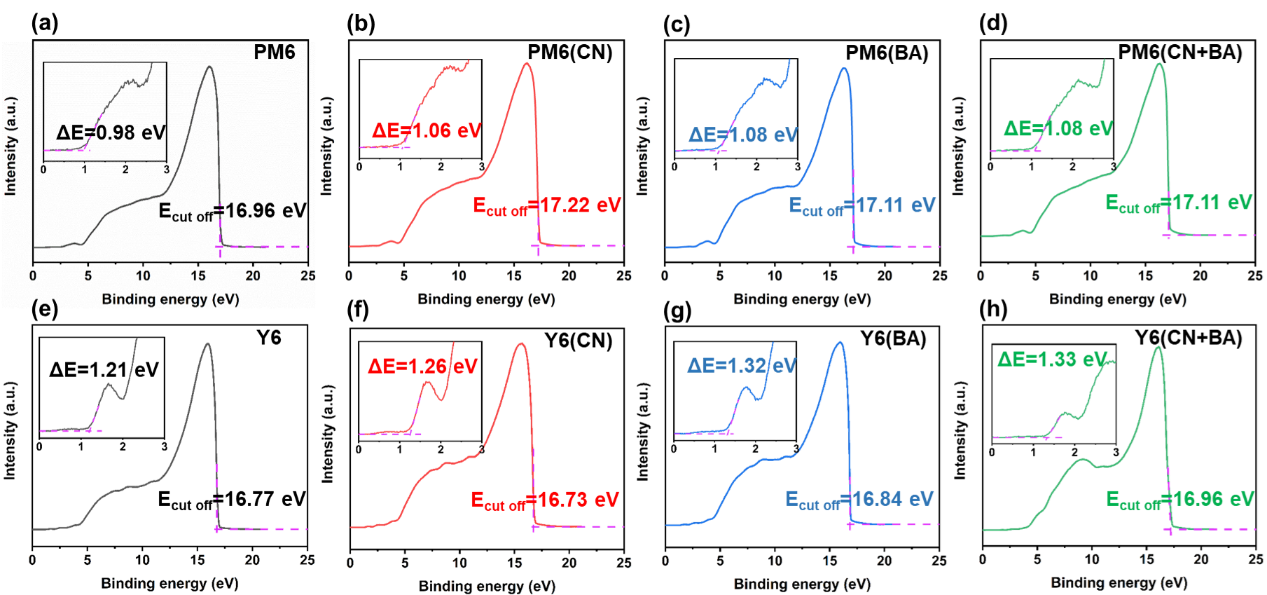


**Figure S2.** Ultraviolet photoelectron spectroscopy spectra for (a-d) PM6 and (e-h) Y6.

**Table S1.** The energy levels of materials were investigated using ultraviolet photoelectron spectroscopy.

| **Treatment** | **LUMO** | **HOMO** | **E_g_** |
| --- | --- | --- | --- |
| **PM6** | -3.41 | -5.24 | 1.83 |
| **PM6(CN)** | -3.23 | -5.06 | 1.83 |
| **PM6(BA)** | -3.36 | -5.19 | 1.83 |
| **PM6(CN+BA)** | -3.36 | -5.19 | 1.83 |
| **Y6** | -4.26 | -5.66 | 1.40 |
| **Y6(CN)** | -4.34 | -5.75 | 1.41 |
| **Y6(BA)** | -4.30 | -5.70 | 1.40 |
| **Y6(CN+BA)** | -4.19 | -5.59 | 1.42 |





**Figure S3**. FT-IR spectra of the BA, control and with BA blend with and without thermal annealing.


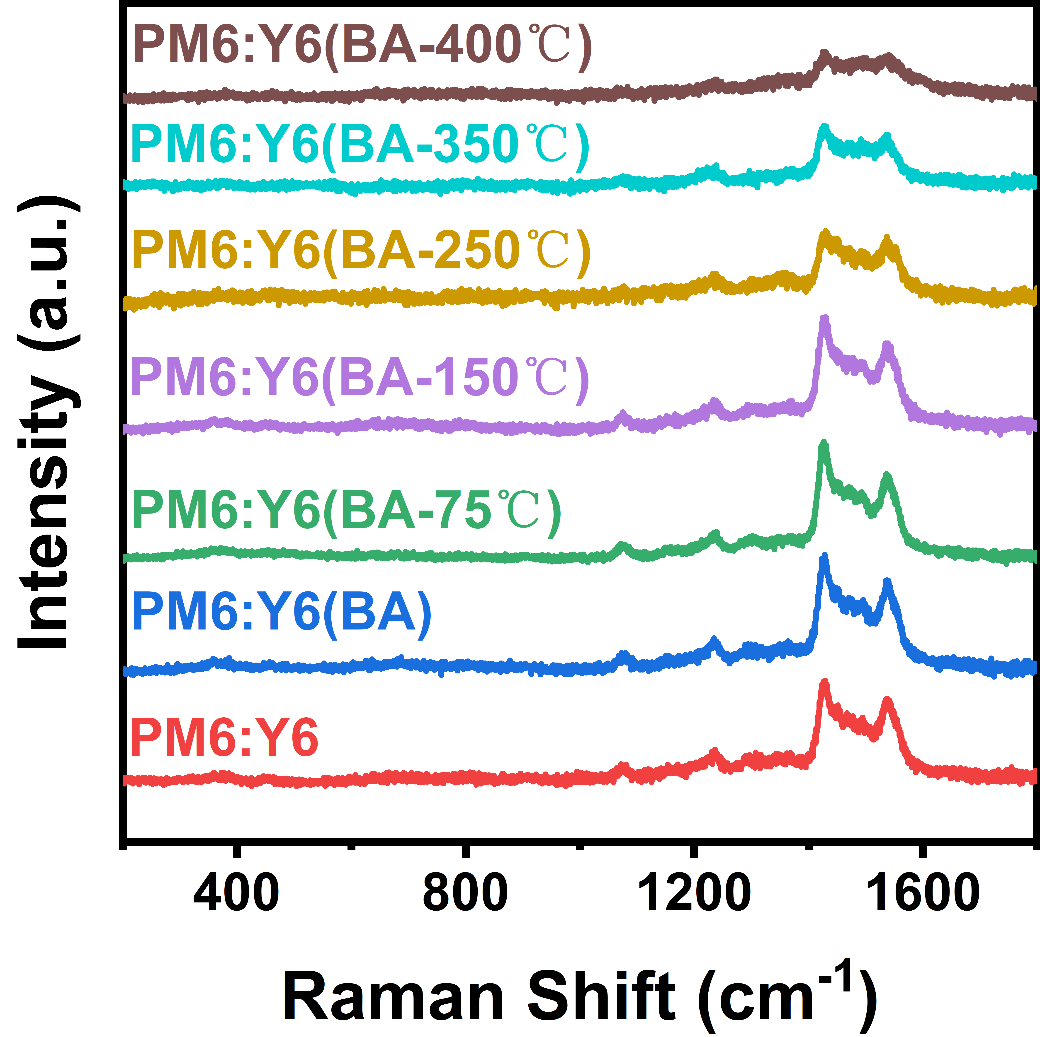


**Figure S4**. Raman spectroscopy of control and with BA blend with and without thermal annealing.


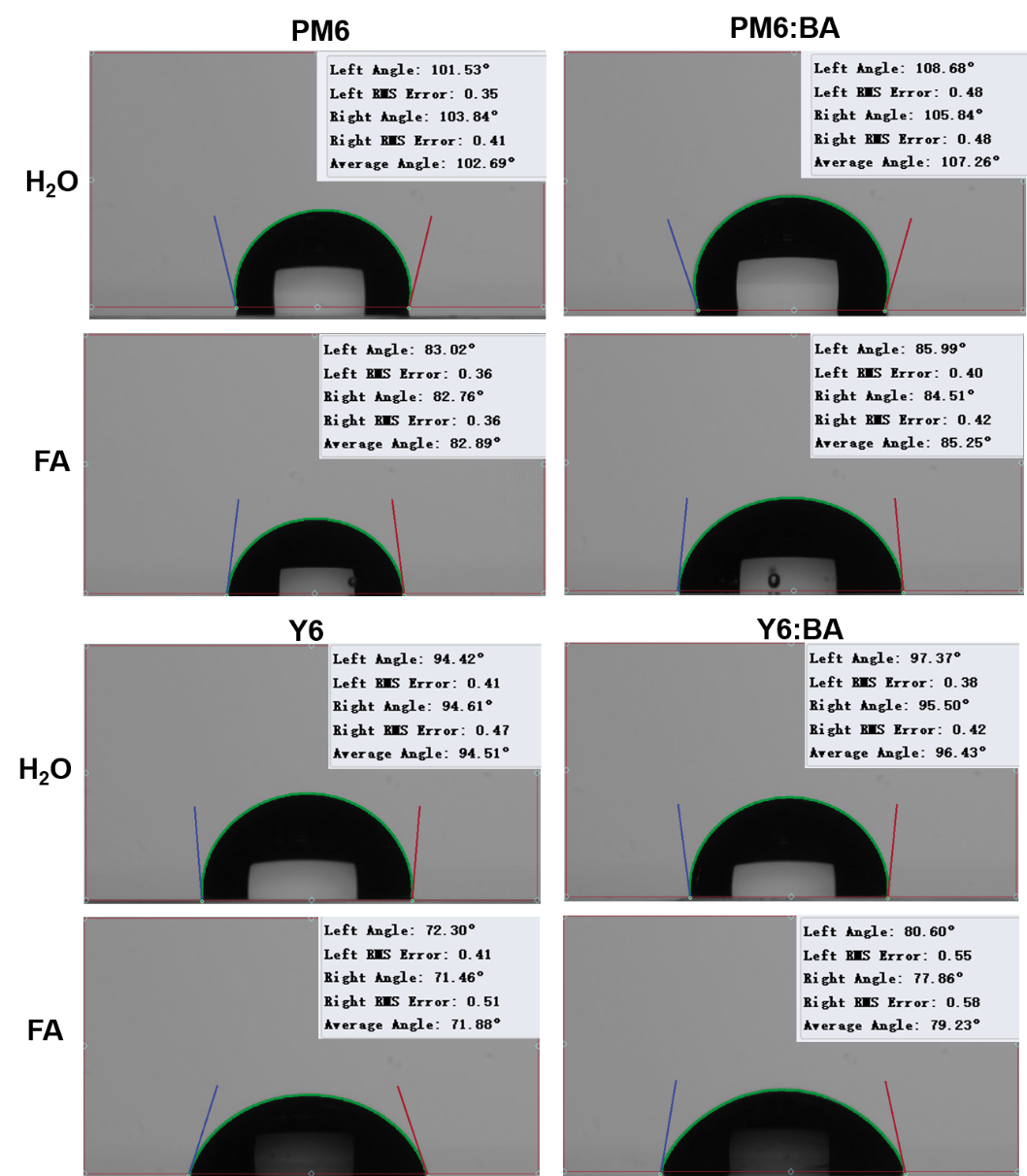


**Figure S5**. Contact angle images of PM6, PM6:BA, Y6, and Y6:BA films with H_2_O and formamid (FA) droplet on top.

**Table S2**. Contact angle, film surface energy between BA and PM6, Y6.

|  | Average | |  | | | surface free energy |
| --- | --- | --- | --- | --- | --- | --- |
|  | H_2_O(θ_1_) | FA(θ_2_) | | $\sqrt{\boldsymbol{\gamma}_{\mathbf{sv}}^{\mathbf{p}}}$ | $\sqrt{\boldsymbol{\gamma}_{\mathbf{sv}}^{\mathbf{d}}}$ | r_sv_ = (r_sv_^p^+r_sv_^d^)^2^ |
| PM6 | 102.69 | 82.89 | | 1.04 | 4.48 | 21.19 |
| PM6:BA | 107.26 | 85.25 | | 0.99 | 4.33 | 19.75 |
| Y6 | 94.51 | 71.88 | | 1.32 | 5.16 | 28.37 |
| Y6:BA | 96.43 | 79.23 | | 1.69 | 4.33 | 21.59 |


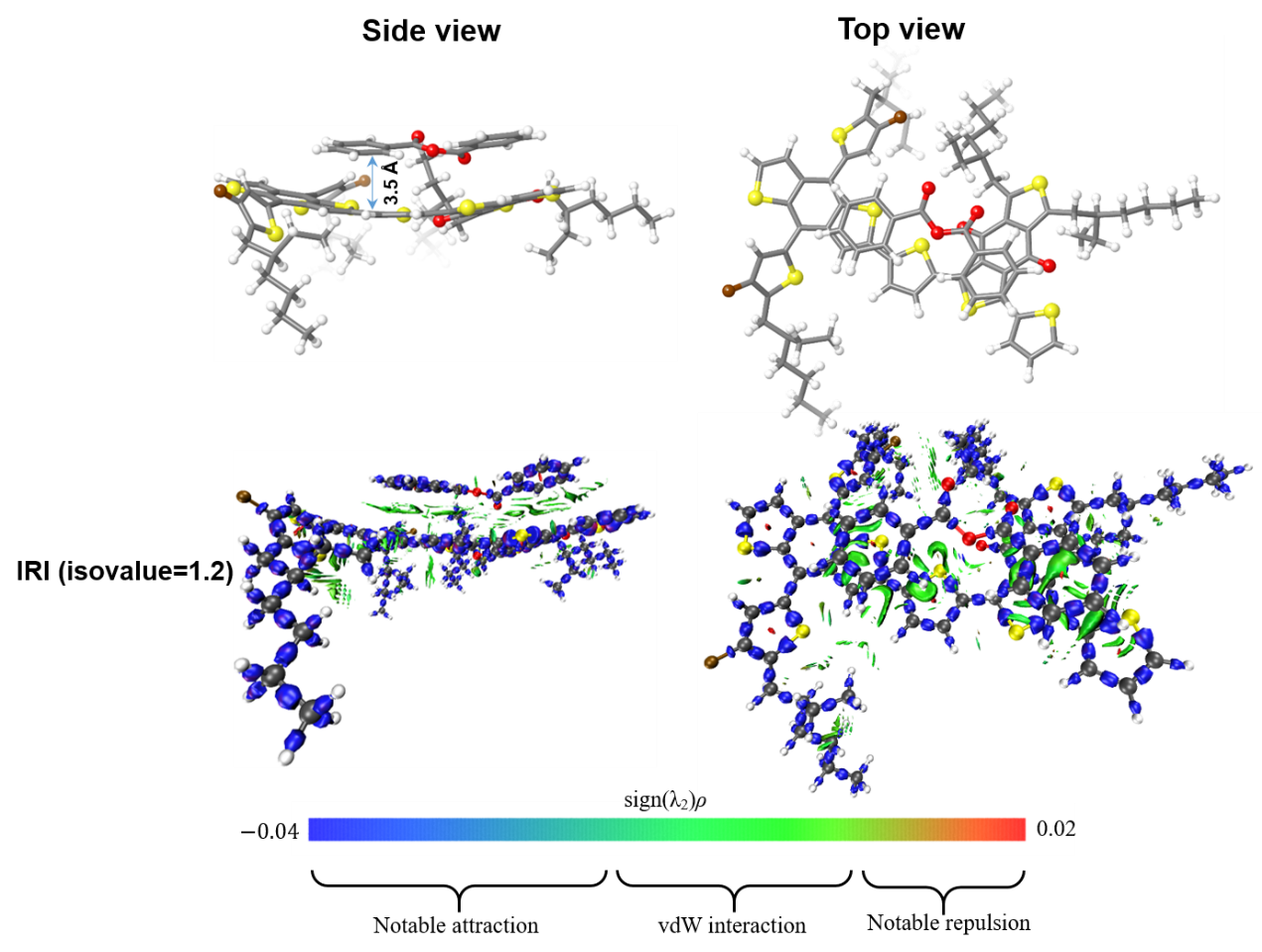


**Figure S6**. The optimized geometry modeled from the semi-empirical xtb program of BA-PM6 and the calculated isosurface maps of IRI for BA-PM6.


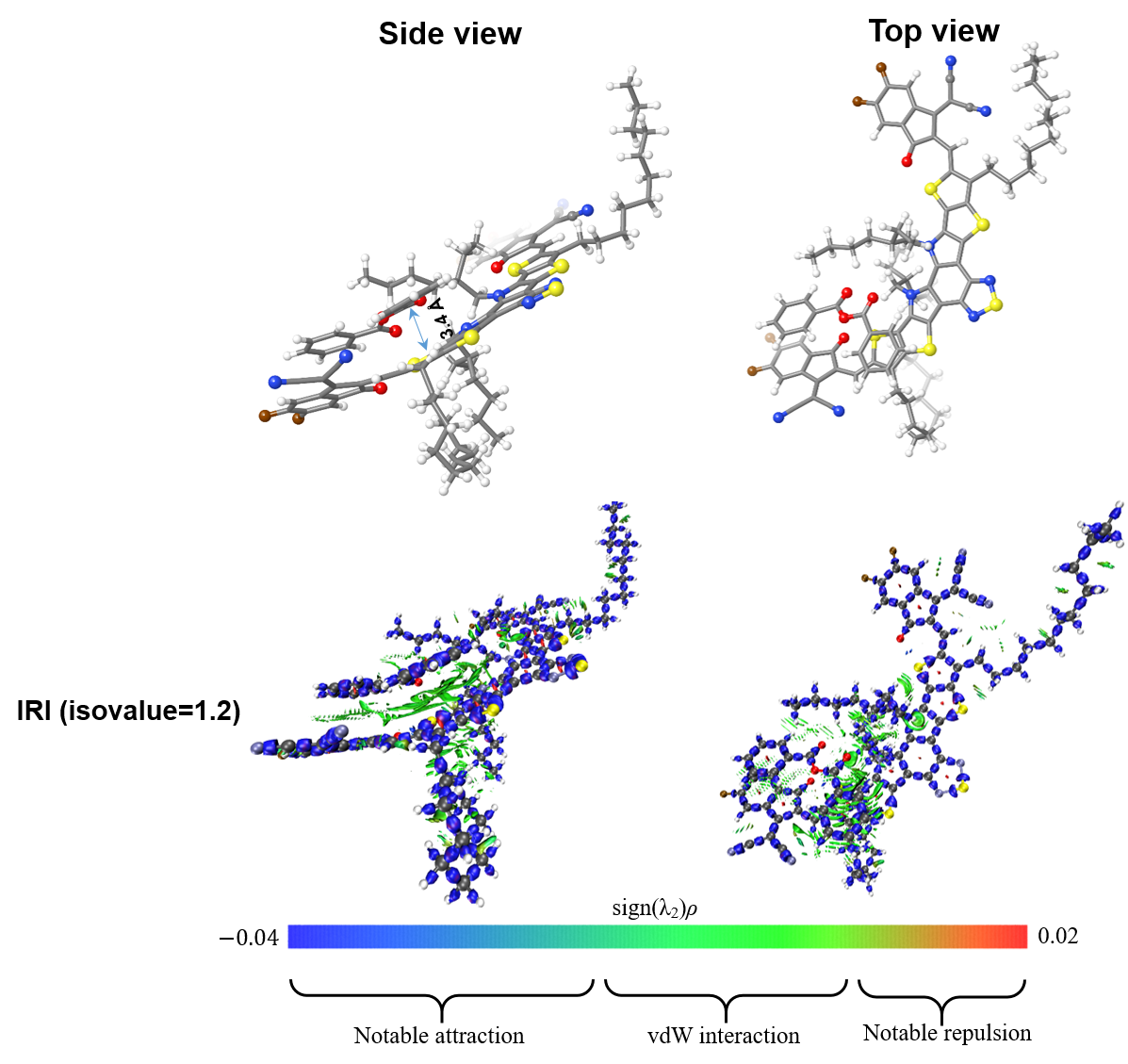


**Figure S7.** The optimized geometry modeled from the semi-empirical xtb program of BA-Y6 end group and the calculated isosurface maps of IRI for BA-Y6 end group.


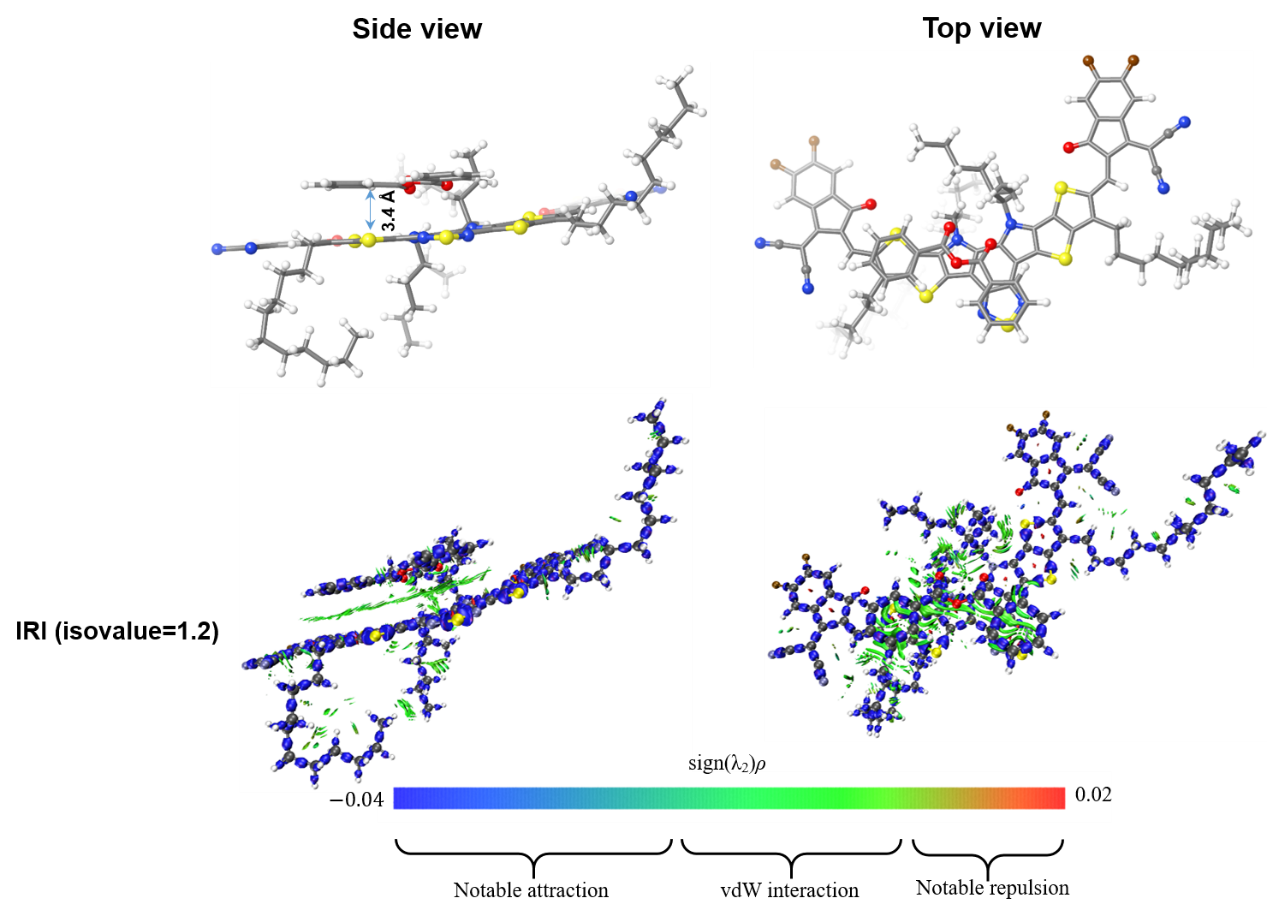


**Figure S8**. The optimized geometry modeled from the semi-empirical xtb program of BA-Y6 central group and the calculated isosurface maps of IRI for BA-Y6 central group.


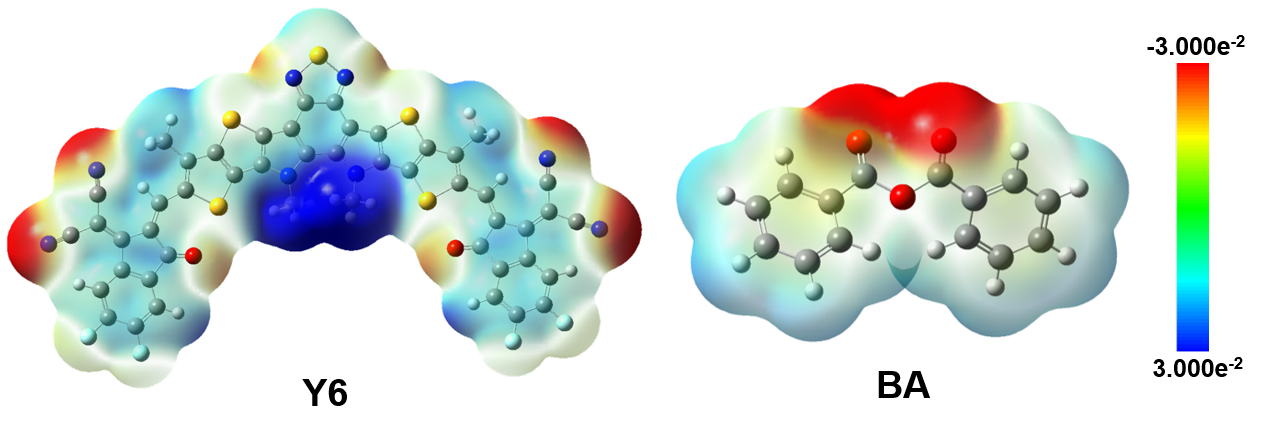


**Figure S9.** The ESP distribution of acceptors Y6 and solid additives BA in this work.


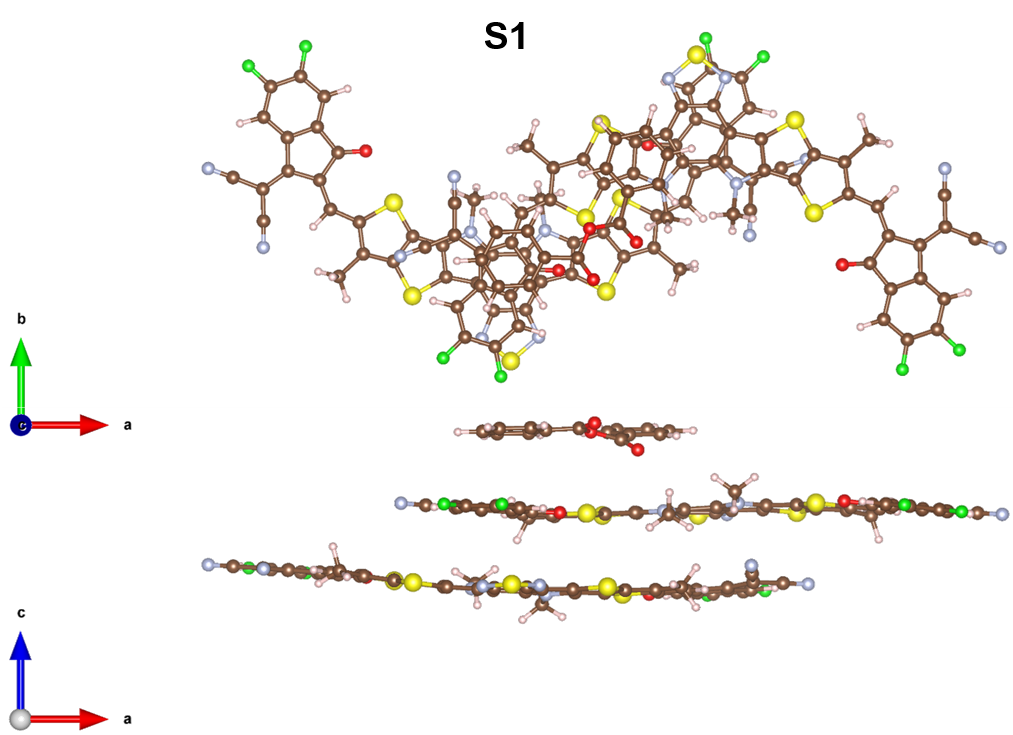


**Figure S10.** The planar and vertical feature of BA adsorb on the Y6 dimer with S1 configuration. The calculated adsorption energy is -0.88 eV.


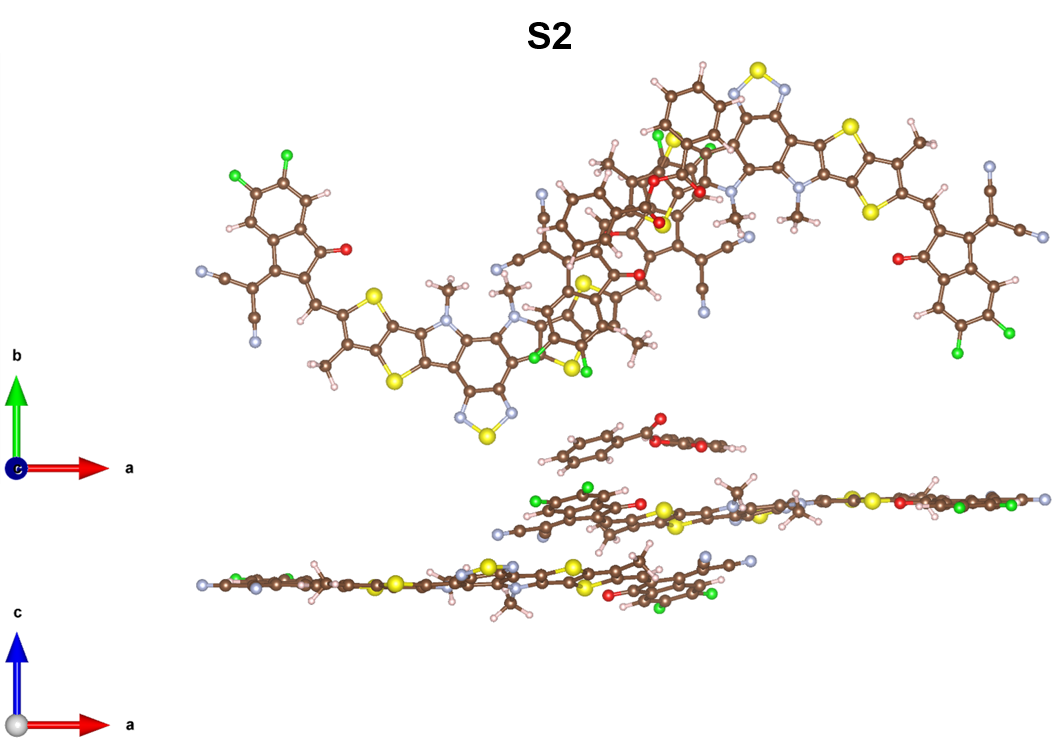


**Figure S11.** The planar and vertical feature of BA adsorb on the Y6 dimer with S2 configuration. The calculated adsorption energy is -1.10 eV.


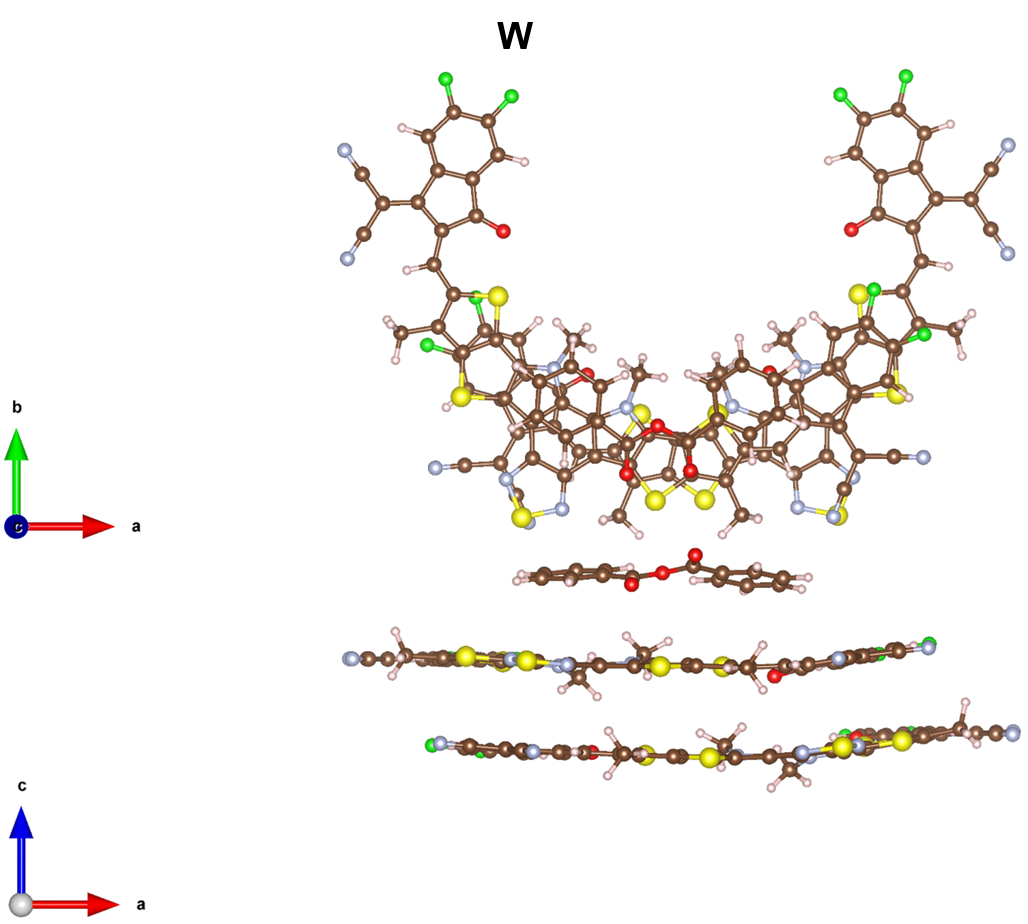


**Figure S12.** The planar and vertical feature of BA adsorb on the Y6 dimer with W configuration. The calculated adsorption energy is -0.78 eV.


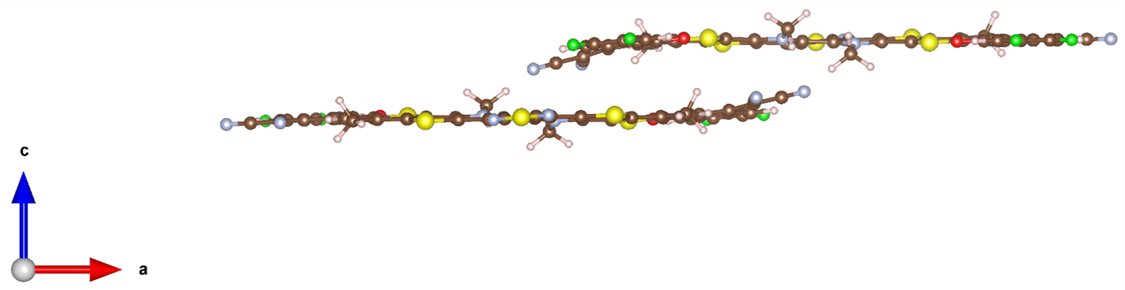


**Figure S13.** The calculated distance between Y6 and Y6, which is 3.63Å.


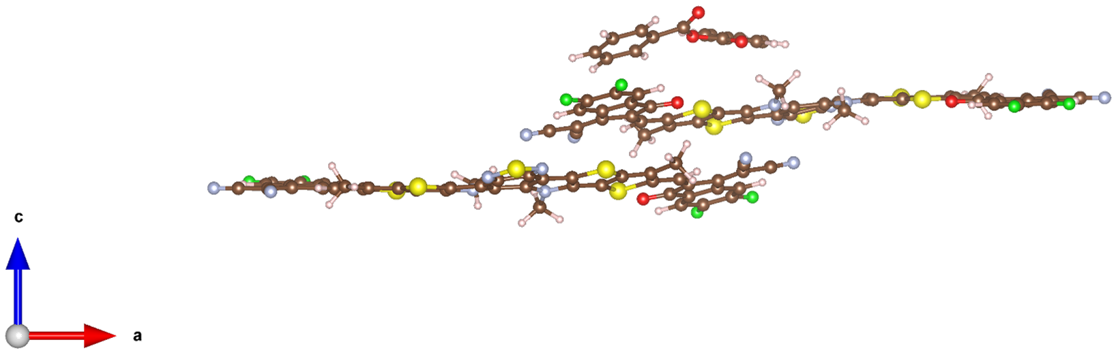


**Figure S14**. The calculated distance between Y6 and Y6 after the BA adsorption, which is 3.59 Å.

**
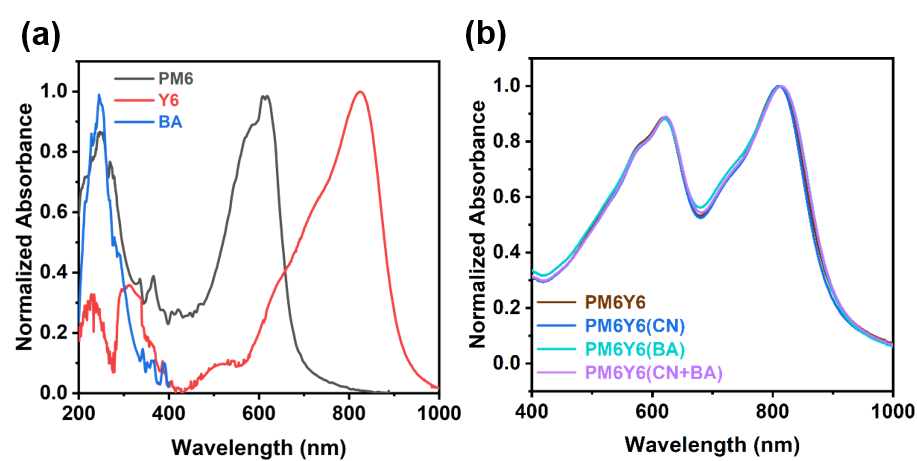
Figure S15.** (a) absorption spectra of PM6, Y6 and BA in films and (b) the normalized absorption spectra of PM6:Y6 films processes with and without additives.


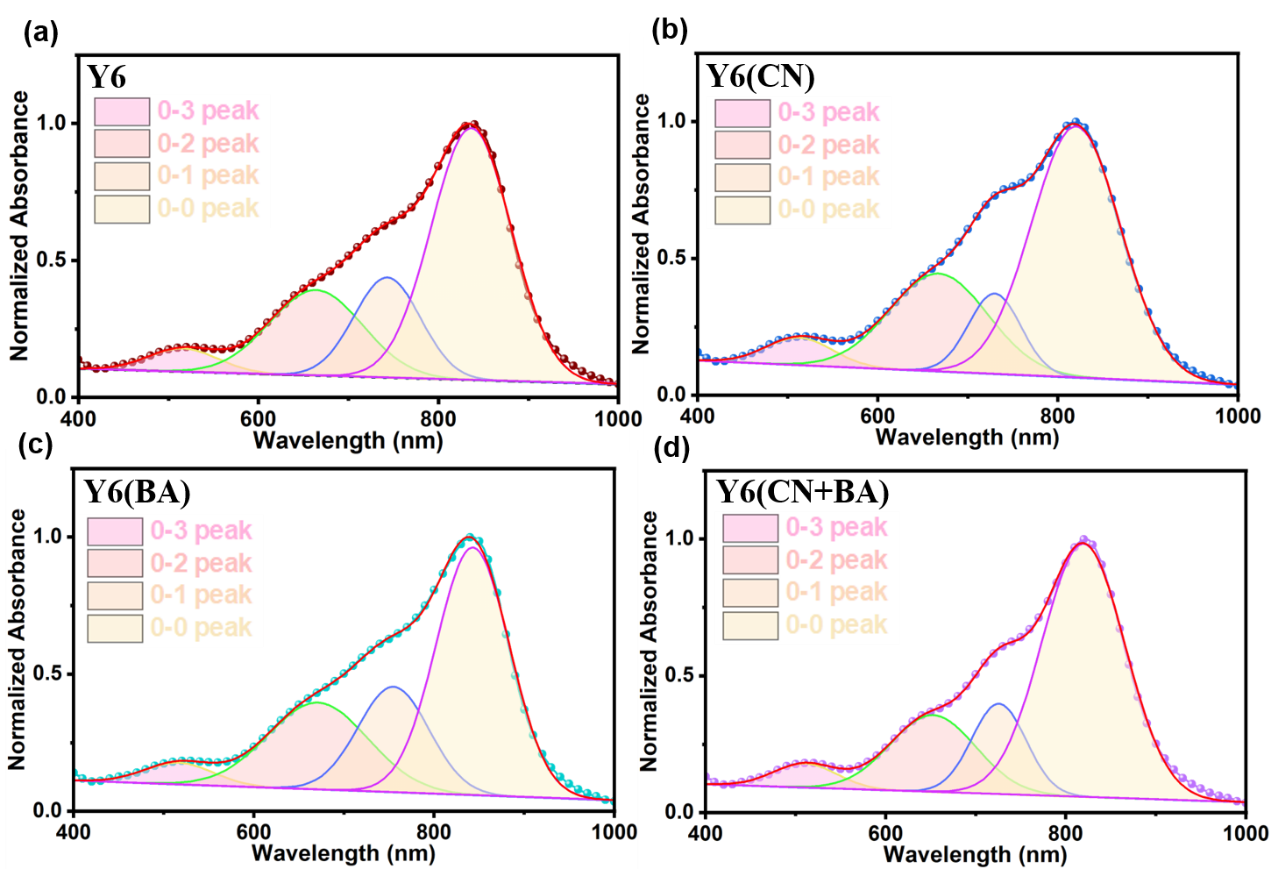


**Figure S16.** (a-d) Fitted absorption spectra of Y6 films processes with and without additives.

**Table S3.** Absorption parameters of Y6 processes with and without additives.

| **Treatment** | **Peak_0-0_** | **A_0-0_/A_0-1_**  **（S_0-0_/S_0-1_）** | **FWHM_0-0_/FWHM_0-1_** |
| --- | --- | --- | --- |
| **Y6** | 837 nm | 2.47 | 1.20 |
| **Y6(CN)** | 820 nm | 3.13 | 1.70 |
| **Y6(BA)** | 843 nm | 2.48 | 1.07 |
| **Y6(CN+BA)** | 819 nm | 2.80 | 1.53 |


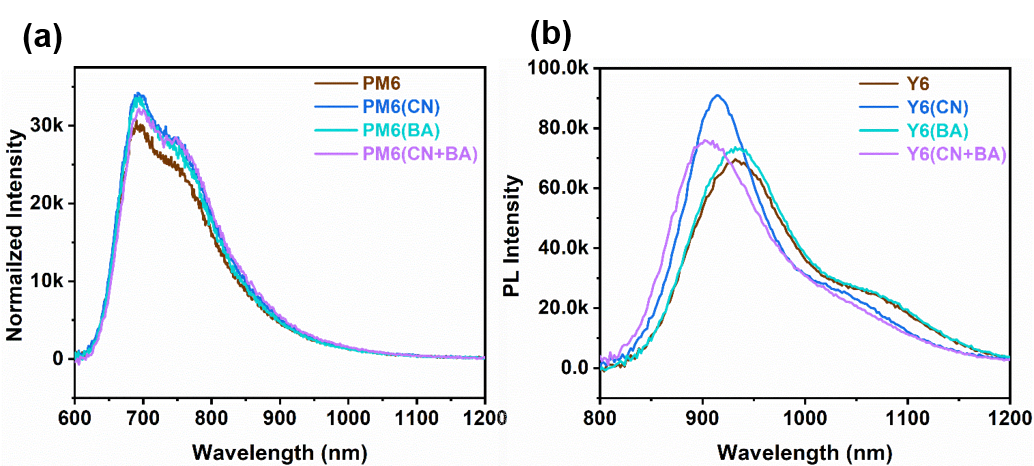


**Figure S17.** PL spectra of (a) PM6 and (b) Y6 films processes with and without additives.


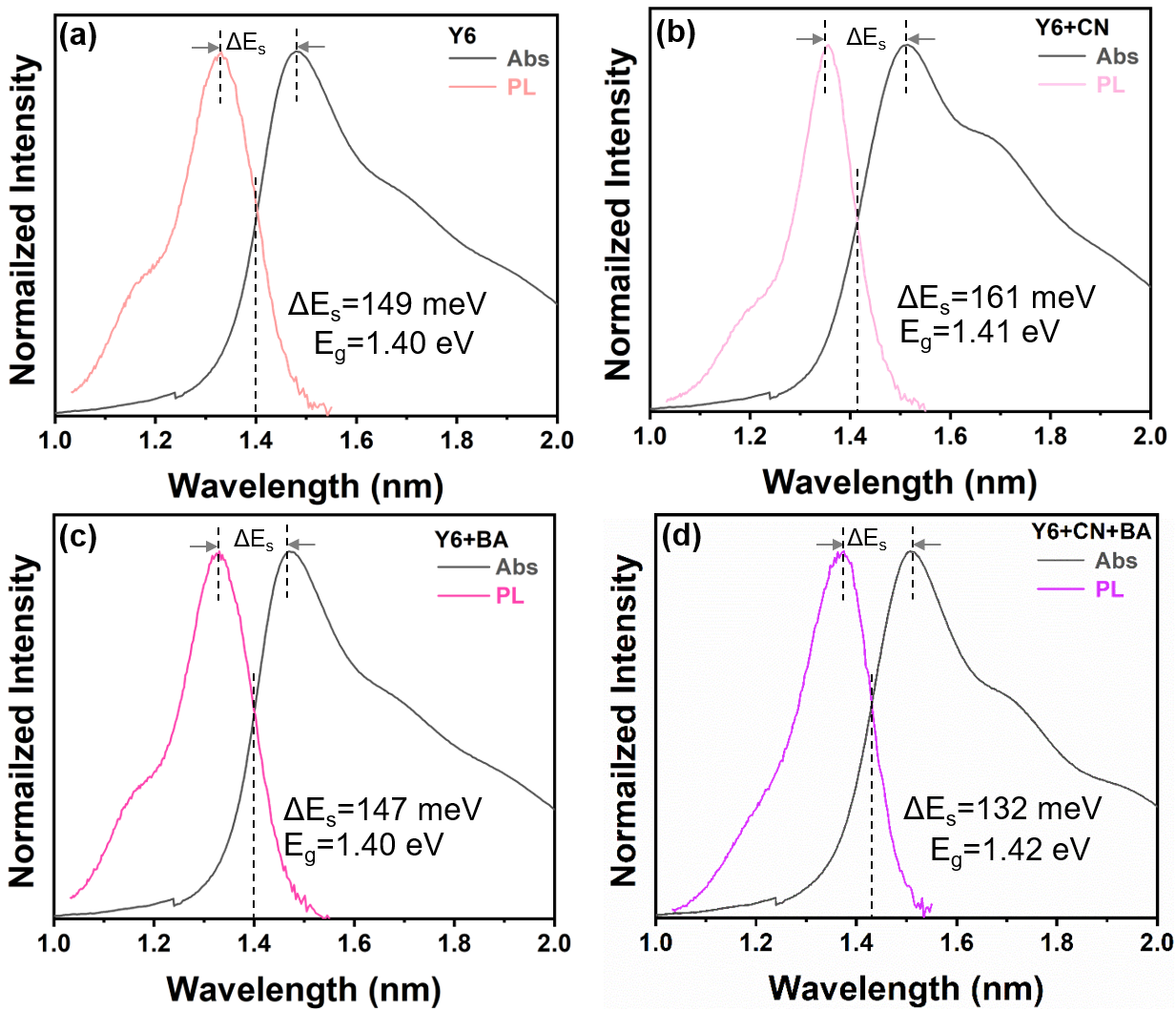


**Figure S18.** Stokes shift (ΔE_S_) of (a) Y6, (b) Y6:CN, (c) Y6:BA and (d) Y6:CN+BA films.


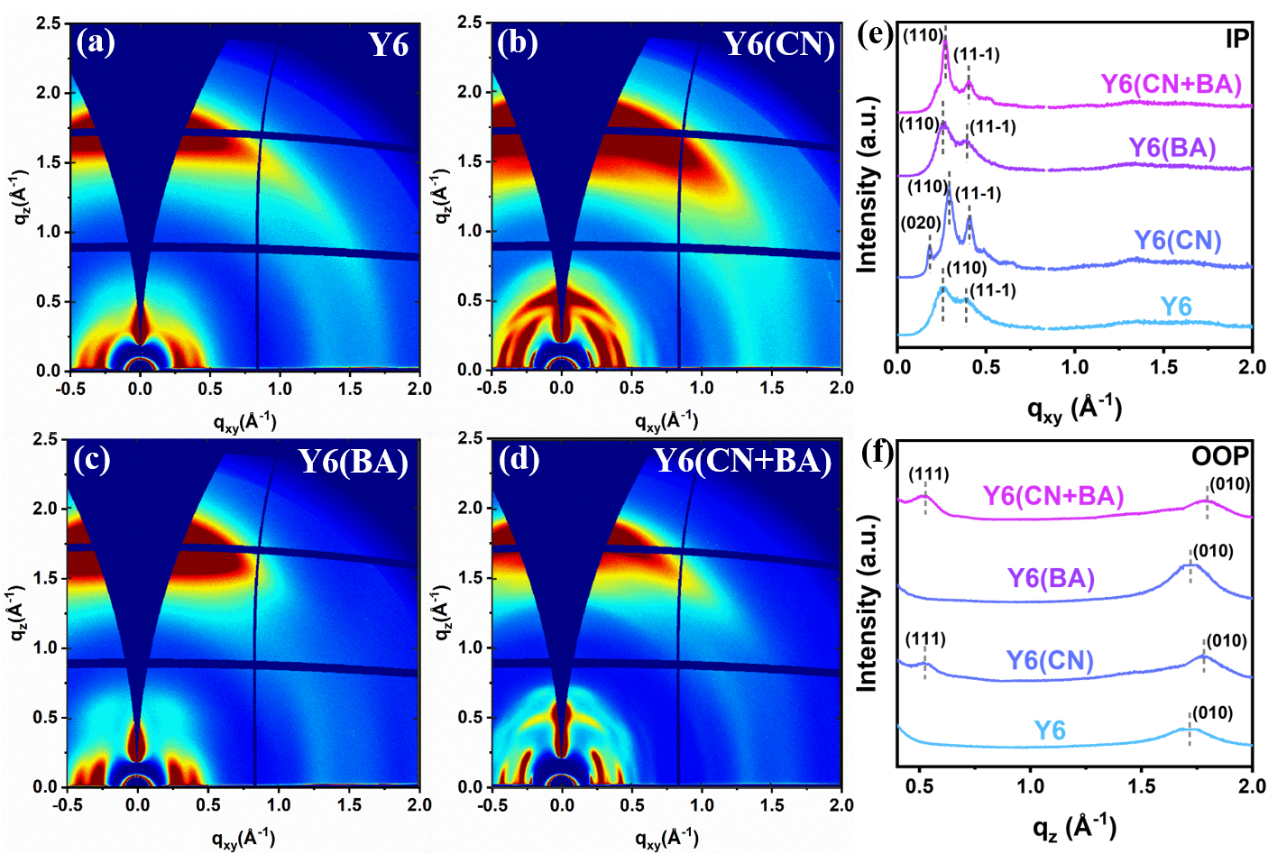


**Figure S19.** 2D GIWAXS patterns of (a) Y6, (b) Y6:CN, (c) Y6:BA and (d) Y6:CN+BA films. (e) The in-plane and (f) out-of-plane line cuts of the Y6 films processes with and without additives.

**Table S4-1.** Calculated parameters for IP directional (110) peak of Y6.

| **Treatment** | **Peak (Å^-1^)** | **d-spacing (Å)** | **FWHM (Å^-1^)** | **CCL (Å)** |
| --- | --- | --- | --- | --- |
| **Y6** | 0.255 | 24.64 | 0.114 | 49.60 |
| **Y6(CN)** | 0.291 | 21.59 | 0.049 | 115.41 |
| **Y6(BA)** | 0.260 | 24.17 | 0.100 | 56.55 |
| **Y6(CN+BA)** | 0.270 | 23.27 | 0.033 | 171.36 |

**Table S4-2.** Calculated parameters for OOP directional (010) peak of Y6.

| **Treatment** | **Peak (Å^-1^)** | **d-spacing (Å)** | **FWHM (Å^-1^)** | **CCL (Å)** |
| --- | --- | --- | --- | --- |
| **Y6** | 1.717 | 3.66 | 0.214 | 26.42 |
| **Y6(CN)** | 1.780 | 3.53 | 0.184 | 30.73 |
| **Y6(BA)** | 1.725 | 3.64 | 0.208 | 27.19 |
| **Y6(CN+BA)** | 1.792 | 3.51 | 0.176 | 32.13 |


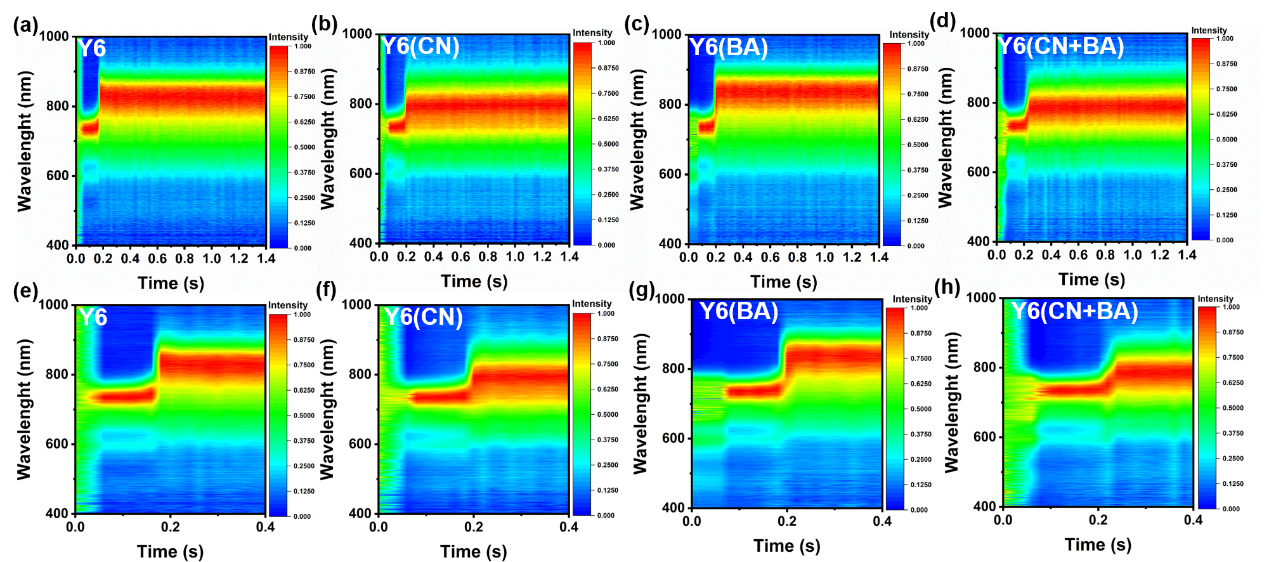


**Figure S20**. In situ absorption of spin-coating process for Y6, Y6:CN, Y6:BA, and Y6:CN+BA.


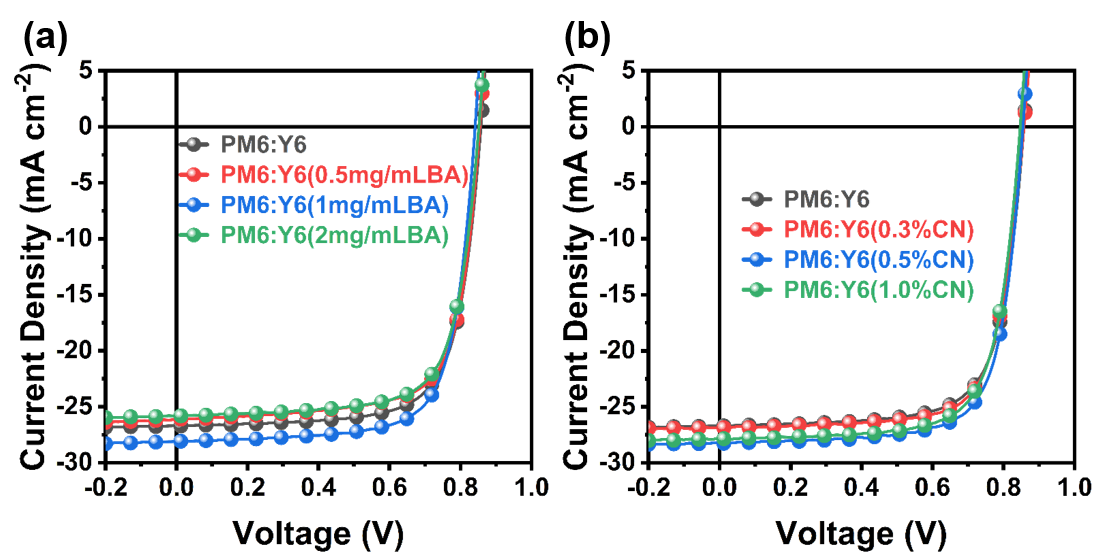


**Figure S21**. J-V curves of the optimal (a) PM6:Y6(BA) and (b) PM6:Y6(CN) devices with different concentrations.

Table S5. The photovoltaic performance in devices with different adding of BA and CN.

| Photoactive layer | V_OC_[V] | J_SC_[mA/cm^2^] | FF[%] | PCE[%] |
| --- | --- | --- | --- | --- |
| PM6:Y6 | 0.86  (0.85±0.005) | 26.70  (27.00±0.43) | 72.45  (71.32±0.91) | 16.57  (16.41±0.12) |
| PM6:Y6  (0.5mg/mLBA) | 0.85  (0.86$\pm$0.006) | 26.13  (25.23$\pm$0.23) | 72.57  (72.90$\pm$0.54) | 16.18  (15.85$\pm$0.35) |
| PM6:Y6  (1mg/mLBA) | 0.84  (0.85$\pm$0.009) | 28.09  (27.64$\pm$0.32) | 73.39  (73.08$\pm$0.82) | 17.33  (17.16$\pm$0.16) |
| PM6:Y6  (2mg/mLBA) | 0.85  (0.85$\pm$0.002) | 25.82  (25.41$\pm$0.33) | 72.48  (71.70$\pm$0.53) | 15.95  (15.55$\pm$0.23) |
| PM6:Y6  (0.3%CN) | 0.86  (0.85$\pm$0.002) | 26.83  (26.13$\pm$1.11) | 73.26  (72.94$\pm$0.57) | 16.85  (16.29$\pm$0.83) |
| PM6:Y6  (0.5%CN) | 0.85  (0.85$\pm$0.001) | 28.24  (28.00$\pm$0.23) | 73.52  (72.99$\pm$1.02) | 17.73  (17.47$\pm$0.24) |
| PM6:Y6  (1%CN) | 0.85  (0.85$\pm$0.003) | 27.82  (27.59$\pm$0.32) | 72.86  (72.76$\pm$0.47) | 17.16  (17.04$\pm$0.10) |


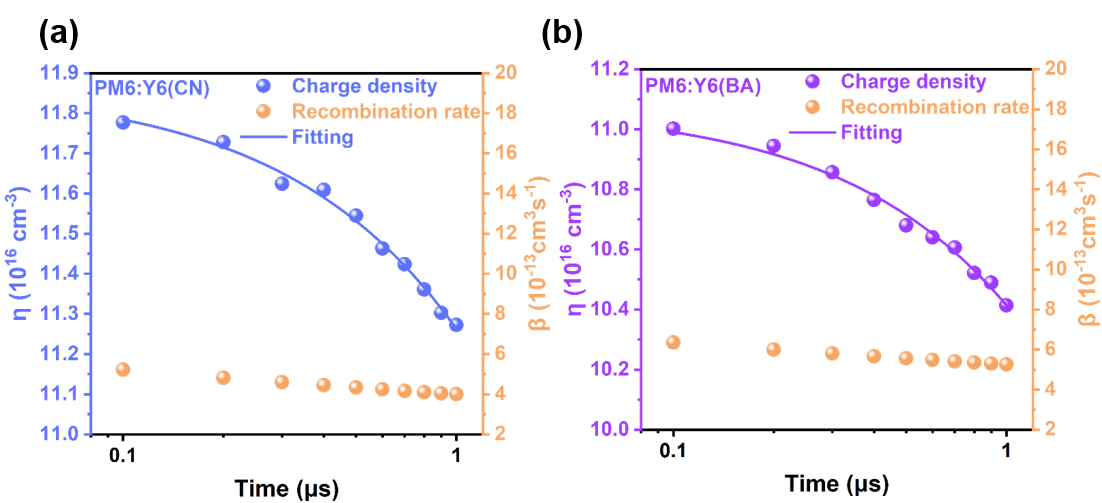


**Figure S22.** Charge extraction and recombination with various delay times of the devices: (a) PM6:Y6(CN) and (b) PM6:Y6(BA).


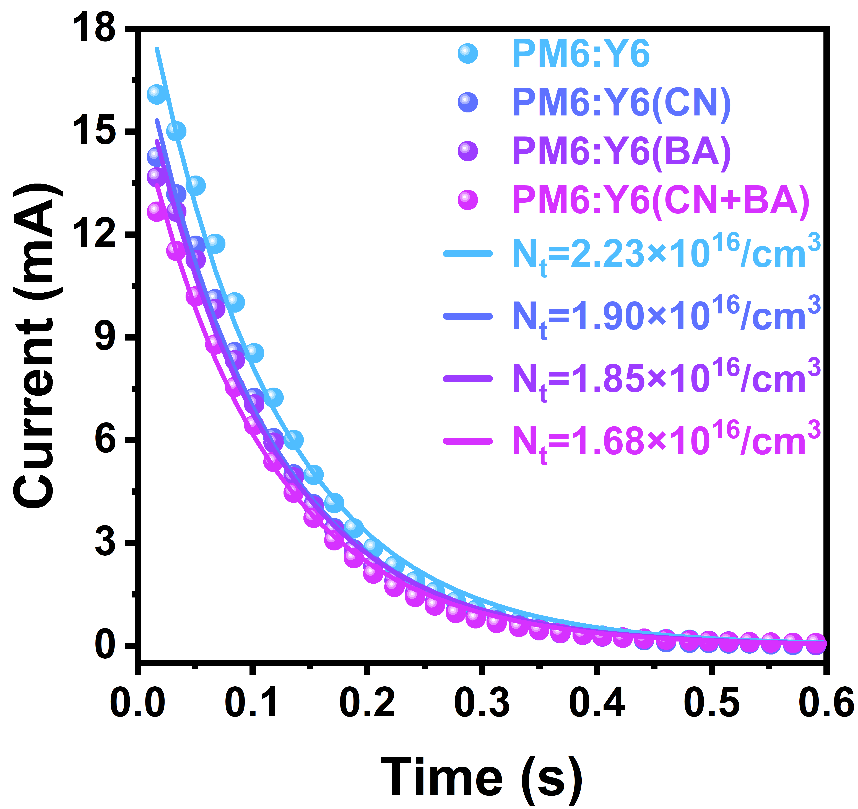


**Figure S23**. Deep-level trap of states (DLTS) of devices based on PM6:Y6 with and without additives.


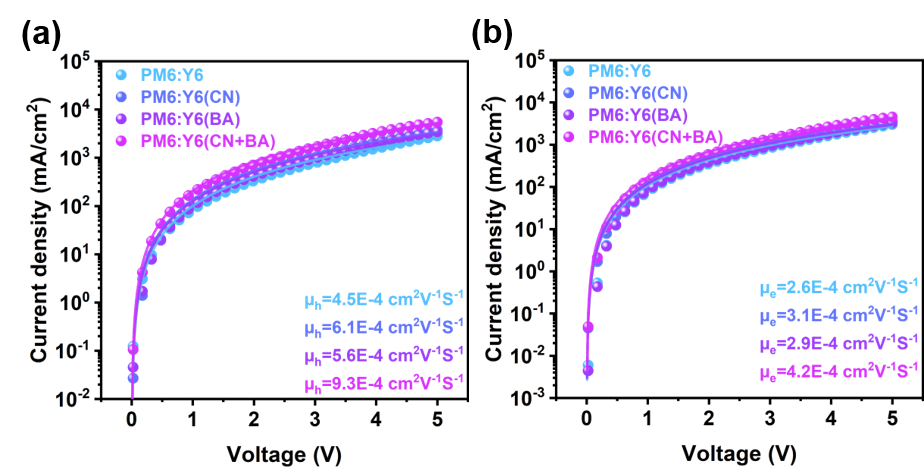


**Figure S24.** (a) μ_h_ and (b) μ_e_: Dark current density vs. applied voltage and SCLC fitting (solid lines) of the experimental data (scatter) of PM6:Y6 processes with and without additives.


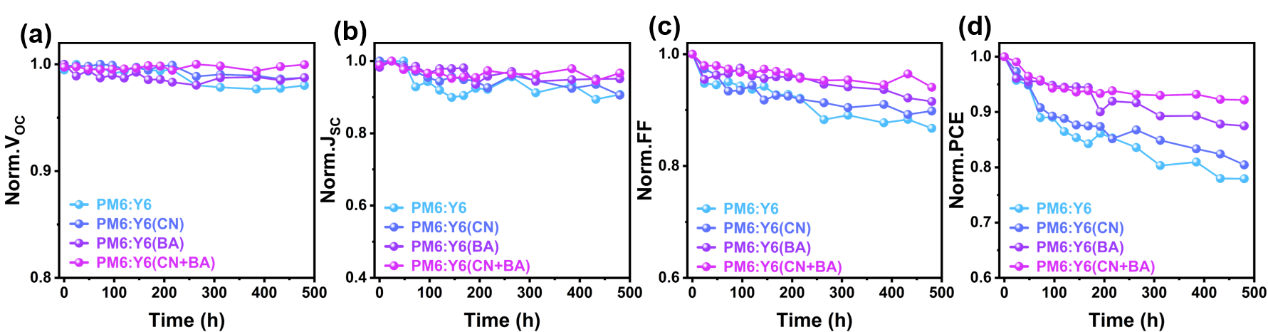


**Figure S25**. Shelf stability of OSCs based on PM6:Y6 with/without additives.


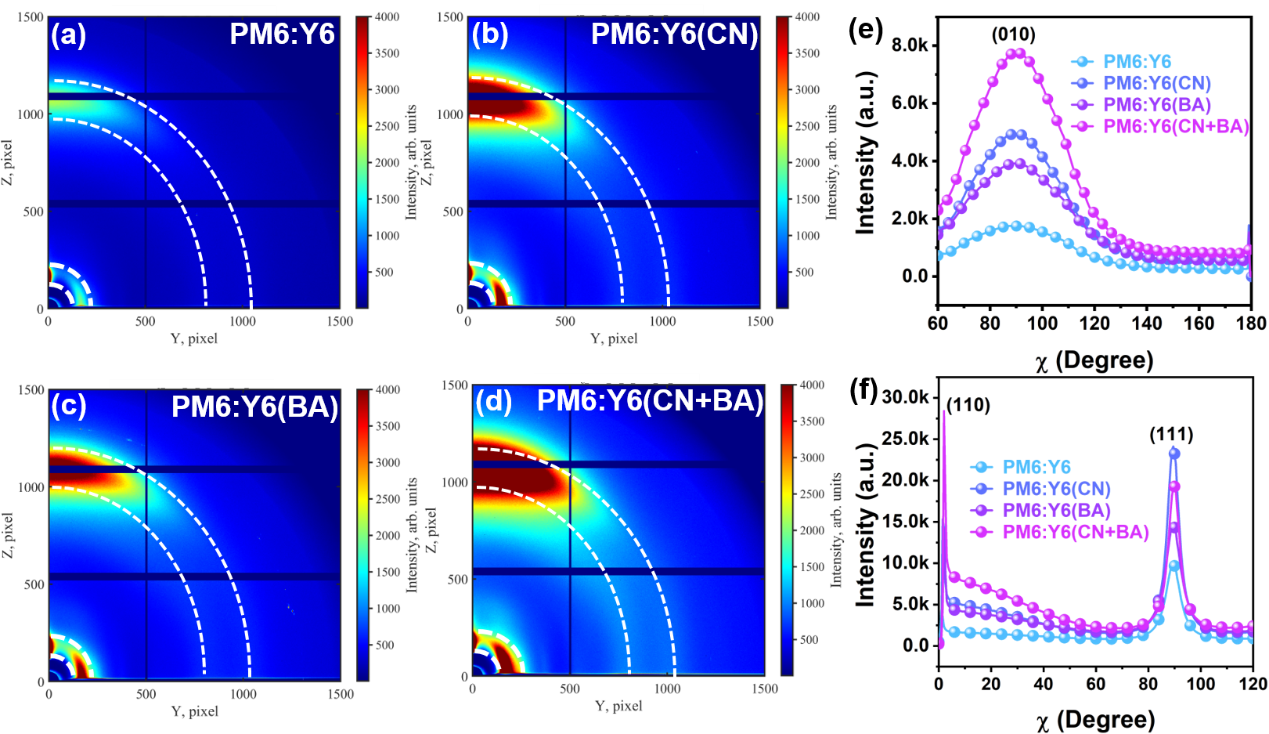


**Figure S26**. (a-d) 2D-GIWAXS pattern of PM6:Y6 films processes with and without additives. The corresponding polar intensity profiles extracted from the (010) (e) and (110), (111) (f) diffraction of PM6:Y6 films processes with and without additives.


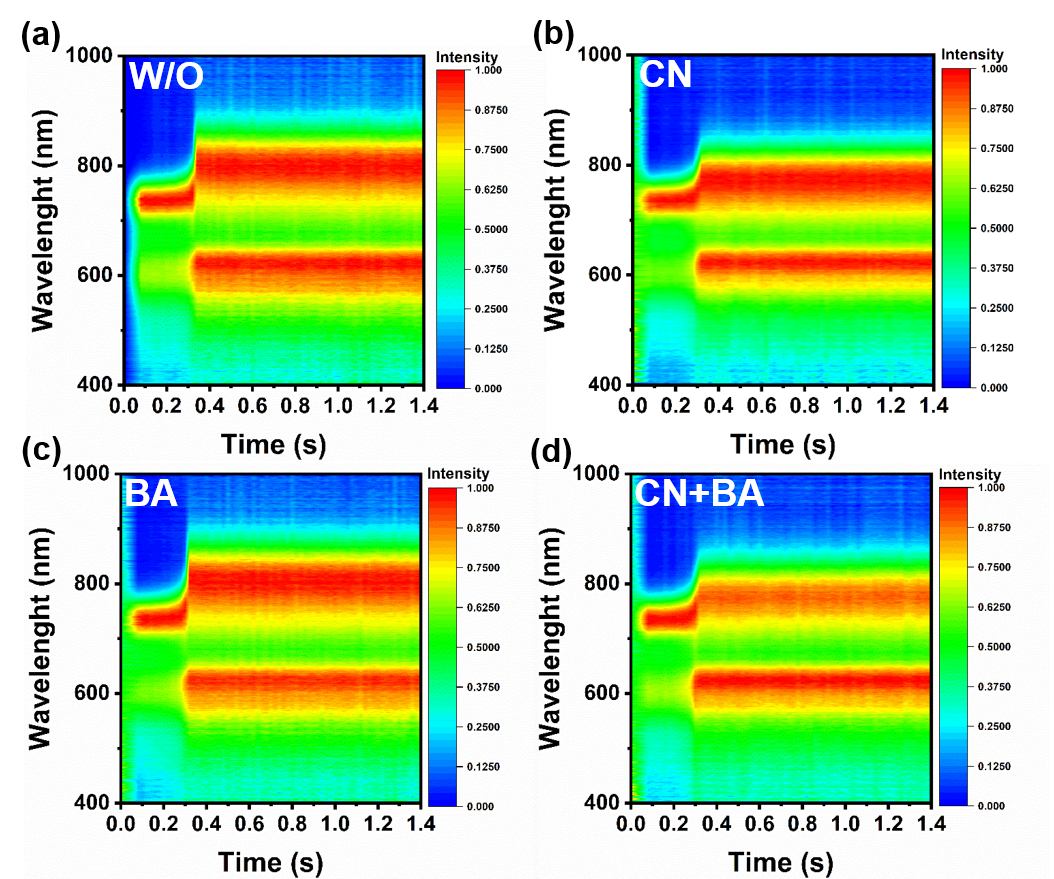


**Figure S27**. In situ absorption of spin-coating process for PM6:Y6, PM6:Y6(CN), PM6:Y6(BA), and PM6:Y6(CN+BA).


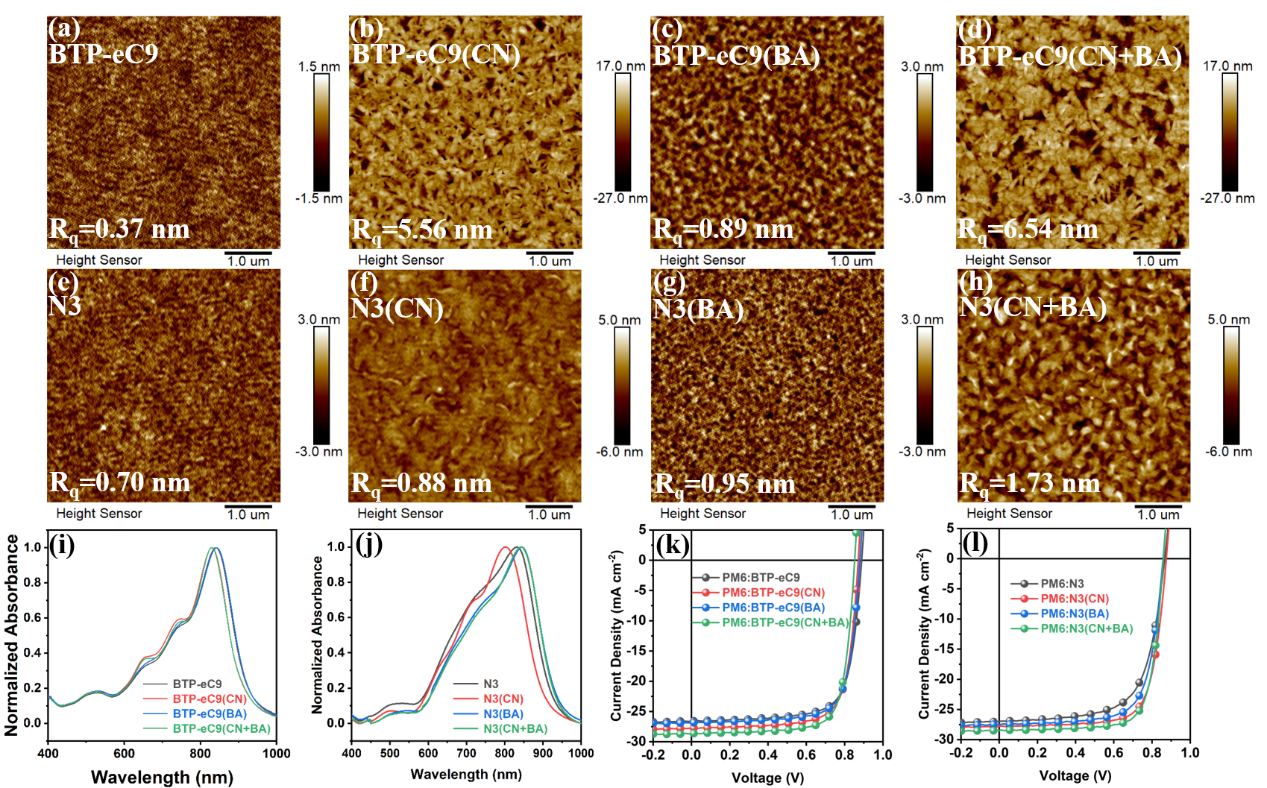


**Figure S28**. AFM height images of (a-d) BTP-eC9 and (e-h) N3 films processes with and without additives. Normalized UV–vis absorption spectra of (i) BTP-eC9 and (j) N3 films processes with and without additives. (k) J-V curves of the optimal PM6: BTP-eC9, PM6: BTP-eC9(CN), PM6: BTP-eC9(BA) and PM6: BTP-eC9 (CN+BA) under the illumination of AM 1.5G irradiance (100 mW cm^-2^). (l) J-V curves of the optimal PM6: N3, PM6: N3(CN), PM6: N3(BA) and PM6: N3(CN+BA) under the illumination of AM 1.5G irradiance (100 mW cm^-2^).

**Table S6-1.** Calculated parameters for IP directional (110) (11-1) peak of PM6:Y6.

| **Treatment** | **Peak** | **Peak (Å^-1^)** | **d-spacing (Å)** | **FWHM (Å^-1^)** | **CCL (Å)** |
| --- | --- | --- | --- | --- | --- |
| **PM6:Y6** | (110) | 0.259 | 24.26 | 0.098 | 57.70 |
|  | (11-1) | 0.330 | 19.04 | 0.238 | 23.76 |
| **PM6:Y6(CN)** | (110) | 0.269 | 23.36 | 0.081 | 69.81 |
|  | (11-1) | 0.328 | 19.16 | 0.225 | 25.13 |
| **PM6:Y6(BA)** | (110) | 0.261 | 24.07 | 0.096 | 58.90 |
|  | (11-1) | 0.328 | 19.16 | 0.227 | 24.91 |
| **PM6:Y6**  **(CN+BA)** | (110) | 0.266 | 23.62 | 0.078 | 72.50 |
|  | (11-1) | 0.330 | 19.04 | 0.211 | 26.80 |

**Table S6-2.** Calculated parameters for OOP directional (010) peak of PM6:Y6.

| **Treatment** | **Peak (Å^-1^)** | **d-spacing (Å)** | **FWHM (Å^-1^)** | **CCL (Å)** |
| --- | --- | --- | --- | --- |
| **PM6:Y6** | 1.706 | 3.68 | 0.214 | 26.42 |
| **PM6:Y6(CN)** | 1.709 | 3.68 | 0.200 | 28.27 |
| **PM6:Y6(BA)** | 1.725 | 3.64 | 0.210 | 26.92 |
| **PM6:Y6**  **(CN+BA)** | 1.706 | 3.68 | 0.198 | 28.56 |

**Table S7.** The photovoltaic parameters of PM6: BTP-eC9 OSCs were measured under AM 1.5G (100 mW cm^-2^) simulated irradiance.

| **Photoactive layer** | **V_OC_ [V]** | **J_SC_ [mA/cm^2^]** | **FF [%]** | **PCE [%]** |
| --- | --- | --- | --- | --- |
| **PM6:BTP-eC9** | 0.89  (0.89$\pm$0.005) | 26.64  (26.24$\pm$0.53) | 73.77  (73.15$\pm$0.57) | 17.47  (17.01$\pm$0.34) |
| **PM6:BTP-eC9(CN)** | 0.87  (0.87$\pm$0.002) | 27.77  (27.22$\pm$0.41) | 74.78  (74.76$\pm$0.25) | 18.08  (17.71$\pm$0.25) |
| **PM6:BTP-eC9(BA)** | 0.88  (0.87$\pm$0.005) | 26.87  (26.62$\pm$0.17) | 74.71  (74.15$\pm$0.42) | 17.67  (17.26$\pm$0.30) |
| **PM6:BTP-eC9(CN+BA)** | 0.85  (0.86$\pm$0.009) | 28.67  (28.27$\pm$0.29) | 76.02  (75.30$\pm$1.00) | 18.59  (18.34$\pm$0.19) |

**Table S8.** The photovoltaic parameters of PM6: N3 OSCs were measured under AM 1.5G (100 mW cm^-2^) simulated irradiance.

| **Photoactive layer** | **V_OC_ [V]** | **J_SC_ [mA/cm^2^]** | **FF [%]** | **PCE [%]** |
| --- | --- | --- | --- | --- |
| **PM6:N3** | 0.87  (0.86$\pm$0.003) | 26.98  (26.32$\pm$0.93) | 66.84  (66.10$\pm$0.79) | 15.64  (15.02$\pm$0.54) |
| **PM6:N3(CN)** | 0.87  (0.87$\pm$0.003) | 27.79  (27.28$\pm$0.39) | 73.93  (73.43$\pm$0.83) | 17.98  (17.44$\pm$0.43) |
| **PM6:N3(BA)** | 0.86  (0.85$\pm$0.002) | 27.49  (27.37$\pm$0.64) | 71.91  (69.82$\pm$1.37) | 16.96  (16.31$\pm$0.44) |
| **PM6:N3**  **(CN+BA)** | 0.86  (0.86$\pm$0.001) | 28.46  (28.14$\pm$0.39) | 75.67  (75.46$\pm$0.68) | 18.52  (18.29$\pm$0.17) |

**References**

1. J. Lv, H. Tang, J. Huang, C. Yan, K. Liu, Q. Yang, D. Hu, R. Singh, J. Lee, S. Lu, G. Li, Z. Kan, Additive-induced miscibility regulation and hierarchical morphology enable 17.5% binary organic solar cells, Energy Environ. Sci. 14 (2021) 3044-3052.
2. X. Meng, C. H. Y. Ho, S. Xiao, Y. Bai, T. Zhang, C. Hu, H. Lin, Y. Yang, S. K. So, S. Yang, Molecular design enabled reduction of interface trap density affords highly efficient and stable perovskite solar cells with over 83% fill factor, Nano Energy 52 (2018) 300.
3. Pivrikas, G. Juska, A. J. Mozer, M. Scharber, K. Arlauskas, N. S. Sariciftci, H. Stubb, R. Osterbacka, Bimolecular recombination coefficient as a sensitive testing parameter for low-mobility solar-cell materials, PhysRevLett, 94(2005) 176806.
4. Liu, Y. Gao, B. Xu, P. H. M. van Loosdrecht, W. Tian, Trap-limited bimolecular recombination in poly(3-hexylthiophene): Fullerene blend films Organic Electronics, 38 (2016) 8.
5. Ni, Z., Bao, C., Liu, Y., Jiang, Q., Wu, W., Chen, S., Dai, X., Chen, B., Hartweg, B., Yu, Z., Holman, Z., Huang, J. Resolving spatial and energetic distributions of trap states in metal halide perovskite solar cells. Science. 2020, 367(6484), 1352–1358.
6. Jiang, K., Wei, Q., Lai, J. Y. L., Peng, Z., Kim, H. K., Yuan, J., Ye, L., Ade, H., Zou, Y., Yan, H. Alkyl chain tuning of small molecule acceptors forefficient organic solar cells. Joule. 2019, 3, 3020-3033.
7. Laudari, A., Guha, S. Temperature dependent carrier mobility in organic fieldeffect transistors: The role of dielectrics. J. Appl. Phys. 2019, 125(3), 035501.
8. Foertig, A., Baumann1, A., Rauh, D., Dyakonov, V., Deibel, C. Charge carrier concentration and temperature dependent recombination in polymer-fullerene solar cells. Appl. Phys. Lett. 2009, 95(5), 052104.
9. Qin, Y., Xu, Y., Peng, Z., Hou, J., Ade, H. Low Temperature Aggregation Transitions in N3 and Y6 Acceptors Enable Double-Annealing Method That Yields Hierarchical Morphology and Superior Efficiency in Nonfullerene Organic Solar Cells. Adv. Funct. Mater. 2020, 30(46), 2005011.
10. Sun, R., Wu, Q., Guo, J., Wang, T., Wu, Y., Qiu, B., Luo, Z., Yang, W., Hu, Z., Guo, 16 / 16 J., Shi, M., Yang, C., Huang, F., Li, Y., Min, J. A Layer-by-Layer Architecture for Printable Organic Solar Cells Overcoming the Scaling Lag of Module Efficiency. Joule. 2020, 4(2), 407-419.
